# Hematopoietic PI3K**δ** deficiency aggravates murine atherosclerosis through impairment of Tregs

**DOI:** 10.1172/jci.insight.155626

**Published:** 2024-11-22

**Authors:** Mario Zierden, Eva Maria Berghausen, Leoni Gnatzy-Feik, Christopher Millarg, Felix Simon Ruben Picard, Martha Kiljan, Simon Geißen, Apostolos Polykratis, Lea Zimmermann, Richard Julius Nies, Manolis Pasparakis, Stephan Baldus, Chanil Valasarajan, Soni Savai Pullamsetti, Holger Winkels, Marius Vantler, Stephan Rosenkranz

**Affiliations:** 1 Department of Cardiology, Heart Center, Faculty of Medicine and University Hospital Cologne;; 2 Center for Molecular Medicine Cologne (CMMC);; 3 Cologne Cardiovascular Research Center (CCRC);; 4 Institute for Genetics; and; 5 CECAD Research Center, University of Cologne, Cologne, Germany.; 6 Center for Infection and Genomics of the Lung (CIGL), Justus Liebig University, Giessen, Germany.; 7 Department of Lung Development and Remodelling, Max Planck Institute for Heart and Lung Research, Bad Nauheim, Germany.

**Keywords:** Cardiology, Immunology, Atherosclerosis, Signal transduction, T cells

## Abstract

Chronic activation of the adaptive immune system is a hallmark of atherosclerosis. As PI3Kδ is a key regulator of T and B cell differentiation and function, we hypothesized that alleviation of adaptive immunity by PI3Kδ inactivation may represent an attractive strategy counteracting atherogenesis. As expected, lack of hematopoietic PI3Kδ in atherosclerosis-prone *Ldlr*^–/–^ mice resulted in lowered T and B cell numbers, CD4^+^ effector T cells, Th1 response, and immunoglobulin levels. However, despite markedly impaired peripheral pro-inflammatory Th1 cells and atheromatous CD4^+^ T cells, the unexpected net effect of hematopoietic PI3Kδ deficiency was aggravated vascular inflammation and atherosclerosis. Further analyses revealed that PI3Kδ deficiency impaired numbers, immunosuppressive functions, and stability of regulatory CD4^+^ T cells (Tregs), whereas macrophage biology remained largely unaffected. Adoptive transfer of wild-type Tregs fully restrained the atherosclerotic plaque burden in *Ldlr*^–/–^ mice lacking hematopoietic PI3Kδ, whereas PI3Kδ-deficient Tregs failed to mitigate disease. Numbers of atheroprotective B-1 and pro-atherogenic B-2 cells as well as serum immunoglobulin levels remained unaffected by adoptively transferred wild-type Tregs. In conclusion, we demonstrate that hematopoietic PI3Kδ ablation promotes atherosclerosis. Mechanistically, we identified PI3Kδ signaling as a powerful driver of atheroprotective Treg responses, which outweigh PI3Kδ-driven pro-atherogenic effects of adaptive immune cells like Th1 cells.

## Abstract 2

Lack of hematopoietic PI3Kd signaling profoundly impairs atheroprotective Treg responses, which outweigh its effects on proinflammatory Th1 responses, ultimately leading to aggravated murine atherosclerosis.

## Graphical abstract

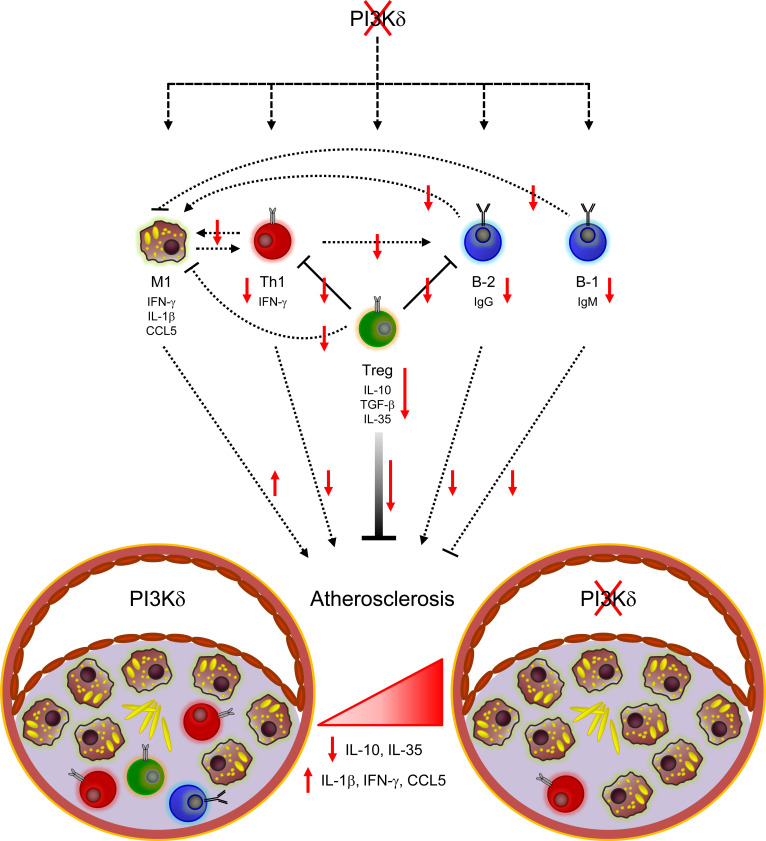

## Introduction

Atherosclerosis is a chronic inflammatory disease driven by subendothelial lipoprotein accumulation, activation of endothelial cells, and intimal leukocyte infiltration of predominantly monocytes and T cells (1). Monocyte-derived macrophages are major effectors of disease through the uptake of lipoproteins, differentiation into lipid-laden foam cells, and secretion of inflammatory mediators (2, 3). Besides innate immune responses, adaptive immunity plays a crucial role in atherosclerosis. Atherosclerosis-prone mice lacking mature T and B lymphocytes develop markedly reduced atheromata (1, 4–6). In particular, IFN-γ–secreting CD4^+^ Th1 cells represent a major cellular component of atherosclerotic plaques, and Th1 cells are key drivers of lesion formation (1, 6, 7). In contrast, regulatory CD4^+^ T cells (Tregs) counterbalance inflammatory processes by immune-suppressive mechanisms (8–10). However, prolonged atherosclerosis possibly leads to impaired stability and atheroprotective functions of Tregs (7, 11, 12). Even though B cells are not numerically prominent in plaque, they are implicated in disease progression (6, 13, 14). Both pro-atherogenic and atheroprotective roles have been assigned to different B cell subsets (13, 14).

Due to the pivotal role of adaptive immunity in atherogenesis, modulating crucial signaling nodes in lymphocytes may represent an attractive strategy to treat atherosclerosis. A wealth of studies revealed that PI3Kδ is a key regulator of adaptive immune responses (15, 16). PI3Kδ is enriched in leukocytes and is activated by core receptors in lymphocyte biology like T cell receptor (TCR), B cell receptor, costimulatory receptor CD28, inducible T cell costimulator (ICOS), as well as IL-2 and IL-4 receptors (15, 16). Upon receptor ligation, PI3Kδ becomes activated and generates phosphatidylinositol 3,4,5-trisphosphate that serves as a membrane docking site for various downstream signaling nodes, including protein kinases AKT and PDK1 (15, 16).

Mice expressing an inactive PI3Kδ isoform (PI3Kδ^D910A/D910A^) show severely impaired Th and T effector (Teff) cell differentiation and function (17, 18). Moreover, PI3Kδ^D910A/D910A^ and PI3Kδ-deficient (PI3Kδ^–/–^) mice display markedly reduced mature B cells, collectively leading to impaired immune responses (19–21). Even though less is known about the role of PI3Kδ in innate immunity (15, 16), it is implicated in proper macrophage differentiation and functions (22, 23).

As a result of the broad effects of PI3Kδ on leukocyte functions, PI3Kδ inactivation attenuates inflammatory diseases such as arthritis, asthma, diabetes, multiple sclerosis, and lupus in mice (15, 16, 24–26). On the other hand, PI3Kδ inactivation can also negatively affect health, causing colitis in mice (15, 22).

While PI3Kδ thus represents an interesting target for the modulation of inflammatory disorders, we investigated its previously unknown role during atherogenesis. To this end, we specifically assessed the role of hematopoietic PI3Kδ on immune mechanisms affecting plaque progression in atherosclerosis-prone LDL receptor–deficient (B6.129S7-*Ldlr^tm1Her^*/J; *Ldlr*^–/–^) mice (27). Since adaptive immunity and particularly Th1 cells are critically involved in the progression of atherosclerosis and PI3Kδ is a key signaling molecule in these lymphocytes, we expected that hematopoietic PI3Kδ deficiency should ameliorate atherogenesis.

However, we demonstrate herein that lack of hematopoietic PI3Kδ signaling affected numbers, functions, and stability of atheroprotective Tregs, which outweighed its impact on pro-inflammatory Th1 responses, ultimately leading to aggravated atherosclerosis.

## Results

### Hematopoietic PI3Kδ deficiency reduces peripheral T and B lymphocytes in Ldlr^–/–^ mice.

To specifically analyze the role of hematopoietic PI3Kδ during atherogenesis while preserving its putative functions in nonimmune cells, lethally irradiated *Ldlr*^–/–^ mice were transplanted with bone marrow (BM) from either PI3Kδ^–/–^ or PI3Kδ^+/+^ mice (henceforth referred to as PI3Kδ^+/+^→*Ldlr*^–/–^ and PI3Kδ^–/–^→*Ldlr*^–/–^ mice). After challenging mice with a high-fat diet (HFD), successful reconstitution with donor BM was verified by representation of donor alleles in peripheral blood cells (Supplemental Figure 1A; supplemental material available online with this article; https://doi.org/10.1172/jci.insight.155626DS1). Furthermore, homogeneous transplantation of CD45.1-expressing mice with CD45.2-expressing PI3Kδ^+/+^ and PI3Kδ^–/–^ BM resulted in very high proportions of donor-derived T and B cells, implying that a similarly high proportion of lymphocytes were of the donor genotype in transplanted *Ldlr*^–/–^ mice (Supplemental Figure 1, B and C).

**Figure 1:**
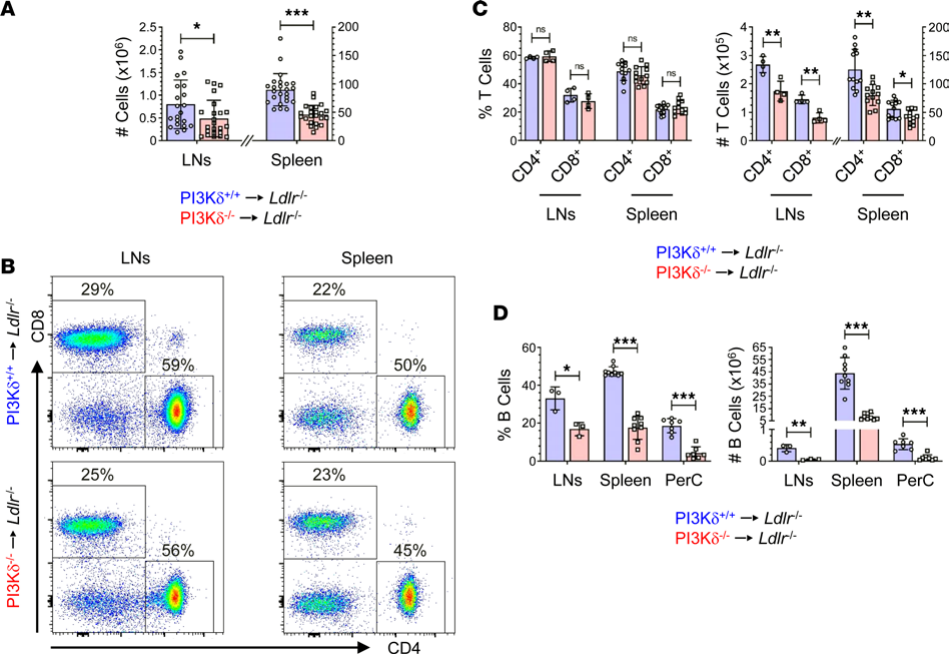
Hematopoietic PI3Kδ deficiency reduces peripheral T and B cells in *Ldlr*^–/–^ mice. Para-aortic lymph nodes (LNs) and spleen of PI3Kδ^+/+^→*Ldlr*^–/–^ (blue) and PI3Kδ^–/–^→*Ldlr*^–/–^ mice (red) were assessed for (**A**) the total number of mononuclear cells (*n* = 21–24), (**B**) CD4 and CD8 expression of T cells, as well as (**C**) proportion and number of CD4^+^ and CD8^+^ T cells (*n* = 4 LNs pooled of 3 mice each, *n* = 12 spleen; see Supplemental Figure 2A for pregating of live CD3^+^NK1.1^–^ T cells). (**D**) Proportion and number of CD19^+^ B cells in spleen, LNs, and peritoneal cavity (PerC; *n* = 9 spleen, *n* = 3 LNs pooled of 3 mice each, *n* = 7 PerC). Statistics were done using 2-tailed unpaired *t* test. *P < 0.05, **P < 0.01, ***P < 0.001.

At first, we investigated the impact of hematopoietic PI3Kδ deficiency on adaptive immunity. HFD-fed PI3Kδ^–/–^→*Ldlr*^–/–^ mice displayed reduced mononuclear cell numbers in para-aortic lymph nodes (LNs) and spleen compared with controls (Figure 1A). CD4^+^ and CD8^+^ T cell numbers in LNs and spleen of PI3Kδ^–/–^→*Ldlr*^–/–^ mice were decreased compared with controls without affecting the relative proportion of these T cell subsets (Figure 1, B and C, and Supplemental Figure 2A). Moreover, CD19^+^ B cells were reduced in spleen, LNs, and peritoneal cavity (PerC) of PI3Kδ^–/–^→*Ldlr*^–/–^ mice (Figure 1D), collectively demonstrating a severe effect of PI3Kδ deficiency on peripheral T and B lymphocytes.

### Hematopoietic PI3Kδ deficiency impairs CD4^+^ Teff cell differentiation and pro-inflammatory Th1 response in Ldlr^–/–^ mice.

**Figure 2:**
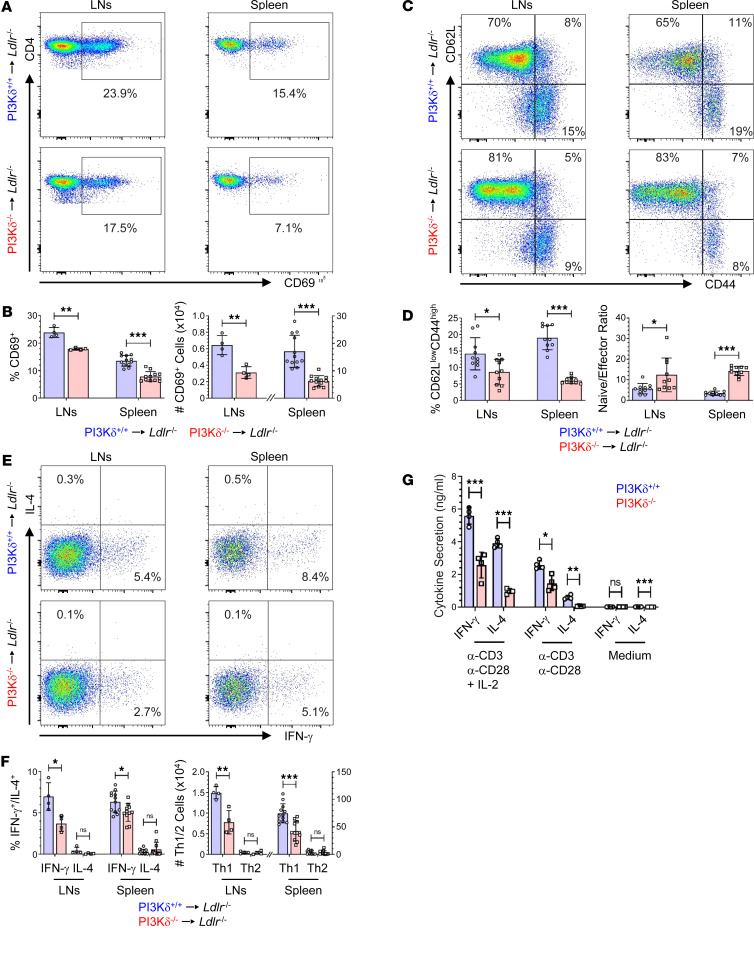
PI3Kδ deficiency impairs CD4^+^ T cell activation, Teff cell differentiation, and pro-inflammatory Th1 response in *Ldlr*^–/–^ mice. Leukocytes from LNs and spleen of PI3Kδ^+/+^→*Ldlr*^–/–^ (blue) and PI3Kδ^–/–^→*Ldlr*^–/–^ mice (red) were pregated for live CD3^+^NK1.1^–^CD4^+^ T cells and investigated for (**A**) CD69 expression, (**B**) proportion and number of CD69^+^ activated cells (*n* = 4 LNs pooled of 3 mice each, *n* = 12 spleen), (**C**) CD62L and CD44 expression, (**D**) proportion of CD62L^lo^CD44^hi^ effector cells and quantification of naive/effector CD4^+^ T cell ratio (*n* = 10), as well as (**E**) intracellular expression of IFN-γ and IL-4 by pregated live CD4^+^ T cells (see Supplemental Figure 2B for gating) and (**F**) proportion and number of IFN-γ^+^ Th1 and IL-4^+^ Th2 cells (*n* = 4 LNs pooled of 3 mice each, *n* = 11–12 spleen). (**G**) Impaired IFN-γ and IL-4 secretion by splenic CD4^+^CD25^–^ T cells from unchallenged PI3Kδ^–/–^ mice upon 72 hours of activation by either anti-CD3/CD28–coated beads in the presence/absence of IL-2 (2,000 U/mL) or by normal medium, respectively (*n* = 4). Statistics were done using 2-tailed unpaired *t* test. *P < 0.05, **P < 0.01, ***P < 0.001.

Since Th1 cells are major drivers of atherogenesis and PI3Kδ is critically involved in Th cell differentiation (1, 6, 7, 17, 18), we investigated the impact of PI3Kδ deficiency on CD4^+^ Teff cell functions in detail. PI3Kδ deficiency led to impaired CD4^+^ T cell activation and Teff cell differentiation in LNs and spleen of PI3Kδ^–/–^→*Ldlr*^–/–^ mice compared with controls (Figure 2, A–D) as measured by expression levels of the CD69 activation marker and of CD44 and CD62L adhesion molecules that delineate T cells into naive (CD62L^hi^CD44^lo^), central memory (CD62L^hi^CD44^hi^), and effector (CD62L^lo^CD44^hi^) cells, respectively. Moreover, hematopoietic PI3Kδ deficiency diminished the Th1 response in secondary lymphoid organs of *Ldlr*^–/–^ mice as assessed by IFN-γ expression (Figure 2, E and F, and Supplemental Figure 2B). Accordingly, splenic CD4^+^CD25^–^ T cells isolated from unchallenged PI3Kδ^–/–^ mice secreted reduced amounts of IFN-γ and IL-4 upon in vitro activation (Figure 2G), demonstrating impaired Th cell functions as a result of PI3Kδ ablation specifically in these cells. However, P3Kδ^–/–^→*Ldlr*^–/–^ mice showed similar CD8^+^ Teff cell differentiation as well as reduced or similar numbers of IFN-γ–expressing cytotoxic CD8^+^ T cells in LNs and spleen as controls (Supplemental Figure 3), respectively.

Thus, hematopoietic PI3Kδ deficiency provokes markedly impaired peripheral CD4^+^ and CD8^+^ T cells, CD4^+^ Teff cell differentiation, and pro-inflammatory Th1 response in *Ldlr*^–/–^ mice.

### Hematopoietic PI3Kδ deficiency exacerbates aortic inflammation and atherosclerosis in Ldlr^–/–^ mice.

**Figure 3:**
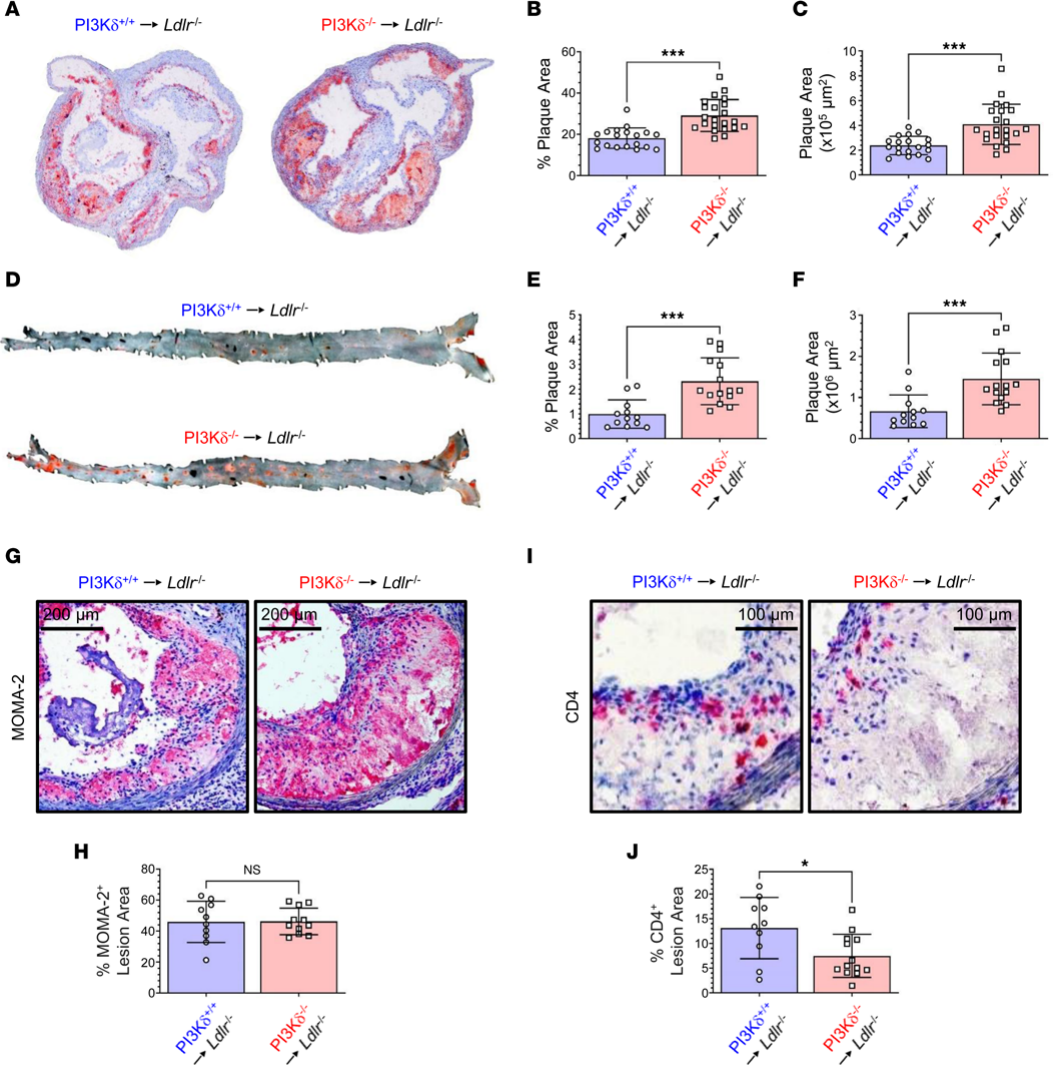
Hematopoietic PI3Kδ deficiency aggravates atherosclerosis in *Ldlr*^–/–^ mice. (**A**) Representative Oil Red O–stained aortic sinus sections and (**B** and **C**) quantification of atherosclerotic lesion size in the aortic sinus of PI3Kδ^+/+^→*Ldlr*^–/–^ (blue) and PI3Kδ^–/–^→*Ldlr*^–/–^ male mice (red) (*n* = 19–22; see Supplemental Figure 4A for quantification of atherosclerotic lesions at each of 4 levels analyzed per mouse). (**D**) Representative Sudan IV–stained en face preparation of whole aortas and (**E** and **F**) quantification of atherosclerotic burden in the whole aorta of male mice (*n* = 12–15). Atherosclerotic lesion areas are depicted as plaque area (μm^2^) and relative plaque area (lesion area as percentage of total vessel area), respectively. Representative aortic sinus sections immunohistochemically stained for (**G**) MOMA-2^+^ macrophages (red) and (**I**) CD4^+^ T cells (red) as well as (**H**) quantification of the macrophage content (*n* = 10–11) and (**J**) CD4^+^ T cell content (*n* = 10–13) in atherosclerotic lesions of indicated male mice. Statistics were done using 2-tailed unpaired *t* test. *P < 0.05, ***P < 0.001.

Unexpectedly, the impairment of the adaptive immune system by hematopoietic PI3Kδ ablation yielded a considerable exacerbation of atherosclerosis in male *Ldlr*^–/–^ mice. Atherosclerotic lesion area and relative lesion area in the aortic roots of male PI3Kδ^–/–^→*Ldlr*^–/–^ mice were increased compared with PI3Kδ^+/+^→*Ldlr*^–/–^ controls (Figure 3, A–C, and Supplemental Figure 4A). Accordingly, atherosclerotic lesions in the whole aorta of male PI3Kδ^–/–^→*Ldlr*^–/–^ mice were enlarged (Figure 3, D–F), though these mice displayed similar body weight, serum lipid, and glucose levels as controls (Supplemental Figure 4B). Surprisingly, female PI3Kδ^–/–^→*Ldlr*^–/–^ mice developed a similar atherosclerotic burden as female controls at similar body weight, serum lipid, and glucose levels (Supplemental Figure 4, C and D), demonstrating that hematopoietic PI3Kδ deficiency exacerbates atherosclerosis in male but not female mice. Hence, we will refer to males in the further course of the study.

**Figure 4:**
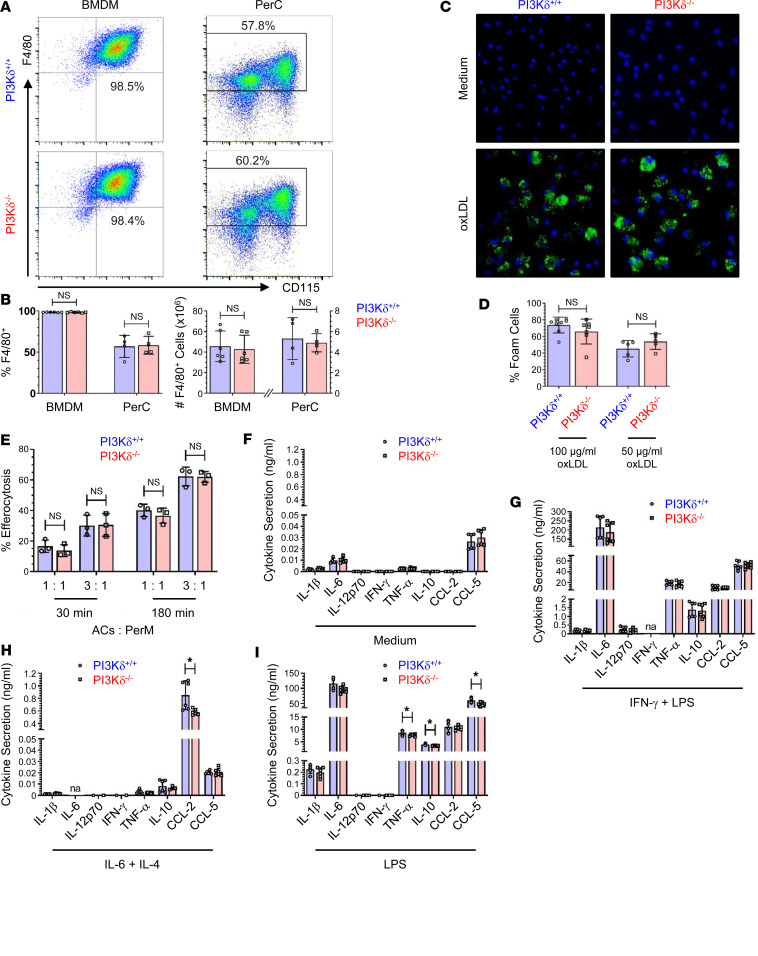
PI3Kδ deficiency has only little effect on monocyte/macrophage biology. BM-derived macrophages (BMDMs) and thioglycollate-induced peritoneal macrophages (PerM) from PI3Kδ^+/+^ (blue) and PI3Kδ^–/–^ mice (red) analyzed for (**A**) F4/80 and CD115 expression as well as (**B**) proportion and numbers of F4/80^+^ cells (*n* = 4–6). (**C**) Representative images of PerM after 48 hours’ incubation with 100 μg/mL oxLDL or control medium stained for lipid droplets by BODIPY 493/503 (green) and nuclei (blue, DAPI), as well as (**D**) quantification of foam cells after oxLDL stimulation (*n* = 5–8). (**E**) Efferocytosis of apoptotic Jurkat T cells (ACs) by PerM at denoted ratios of ACs versus PerM and 30 or 180 minutes of cell culture, respectively (*n* = 3). Data are representative of 4 experiments performed in triplicates. Cytokine secretion by BMDMs upon 24 hours of stimulation with (**F**) control medium, (**G**) 100 ng/mL IFN-γ and 1 μg/mL LPS (classically activated M1 macrophages), (**H**) 50 ng/mL IL-6 followed by 10 ng/mL IL-4 and 50 ng/mL IL-6 (alternatively activated M2 macrophages), and (**I**) 1 μg/mL LPS (*n* = 6). Statistics were done using 2-tailed unpaired *t* test. *P < 0.05; na, not analyzed.

Atheromata of PI3Kδ^–/–^→*Ldlr*^–/–^ mice exhibited a similar macrophage content as controls (Figure 3, G and H), while in accordance with peripheral lymphoid organs, the CD4^+^ T cell content was diminished (Figure 3, I and J). In line with the unchanged atheromatous macrophage content in PI3Kδ^–/–^→*Ldlr*^–/–^ mice, PI3Kδ deficiency did not significantly alter major monocyte/macrophage functions known to contribute to atherogenesis such as monocyte and neutrophil counts in lymphoid organs and blood, as well as differentiation, migration, foam cell formation, and efferocytosis (Figure 4, A–E, and Supplemental Figure 5). Furthermore, PI3Kδ^–/–^ macrophages secreted similar or mildly reduced levels of major cytokines upon in vitro stimulation (Figure 4, F–I). However, since polarization-dependent either both pro-atherogenic and atheroprotective or only pro-atherogenic cytokine secretion was diminished by PI3Kδ ablation (Figure 4, H and I), putative effects on atherogenesis remain elusive.

**Figure 5:**
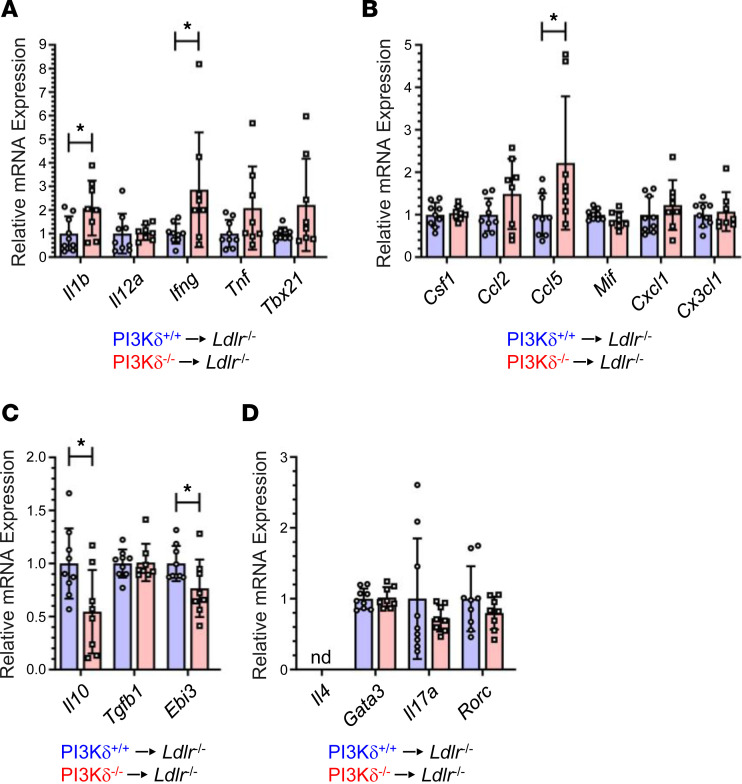
Hematopoietic PI3Kδ deficiency aggravates aortic inflammation in *Ldlr*^–/–^ mice. Relative mRNA expression of (**A**) pro-atherogenic cytokines and transcription factors, (**B**) chemokines, (**C**) atheroprotective cytokines, as well as (**D**) Th2/Th17/M2-related cytokines and transcription factors in aortas of PI3Kδ^+/+^→*Ldlr*^–/–^ (blue) and PI3Kδ^–/–^→*Ldlr*^–/–^ (red) male mice determined by real-time quantitative reverse transcription PCR (qRT-PCR) (*n* = 8–9). Data were normalized to *Gapdh* mRNA expression. Statistics were done using 2-tailed unpaired *t* test. *P < 0.05; nd, not detected.

Aortas of PI3Kδ^–/–^→*Ldlr*^–/–^ mice displayed considerably increased mRNA levels of pro-atherogenic cytokines *Ifng* and *Il1b*, while *Tnf* as well as the Th1 transcription factor *Tbx21* showed a trend toward augmented expression levels (*P* = 0.1 and *P* = 0.09, respectively) compared with PI3Kδ^+/+^→*Ldlr*^–/–^ controls, respectively (Figure 5A). In addition, we found increased mRNA expression of the *Ccl5* chemokine (Figure 5B), which is crucially involved in recruitment of inflammatory Ly6C^hi^ monocytes to atherosclerotic plaques (28, 29). In contrast, aortic mRNA expression of atheroprotective *Il10* and *Ebi3* (subunit of cytokines IL-27 and IL-35) was decreased in PI3Kδ^–/–^→*Ldlr*^–/–^ mice, while *Tgfb1* as well as Th2/Th17/alternatively activated macrophage–associated (M2-associated) cytokines and transcription factors remained unaffected (Figure 5, C and D).

These data collectively demonstrate an increased aortic pro-inflammatory cytokine/chemokine profile and atherosclerotic plaque growth in male PI3Kδ^–/–^→*Ldlr*^–/–^ mice, despite reduced CD4^+^ T cell plaque content and impaired Th1 immunity in secondary lymphoid organs.

### PI3Kδ deficiency impairs numbers, stability, and functions of regulatory T cells aggravating atherosclerosis in Ldlr^–/–^ mice.

**Figure 6:**
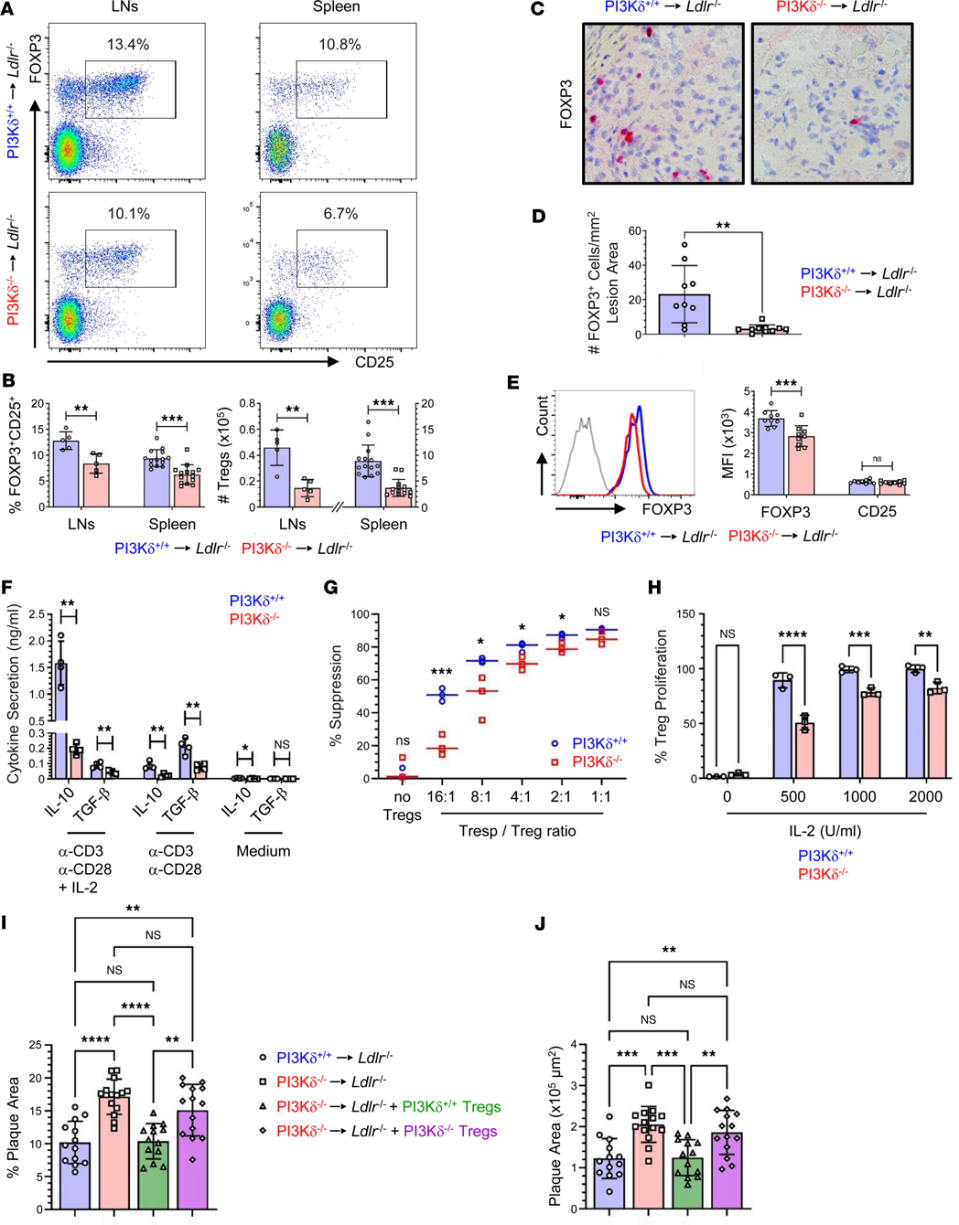
Hematopoietic PI3Kδ deficiency impairs numbers, functions, and stability of Tregs aggravating atherosclerosis in *Ldlr*^–/–^ mice. (**A**) FOXP3 and CD25 expression as well as (**B**) proportion and number of CD25^+^FOXP3^+^ Tregs pregated on live CD4^+^ T cells from LNs and spleen of PI3Kδ^+/+^→*Ldlr*^–/–^ (blue) and PI3Kδ^–/–^→*Ldlr*^–/–^ (red) male mice (*n* = 5 LNs pooled of 3 mice each, *n* = 14 spleen). (**C**) Representative aortic sinus sections immunohistochemically stained for FOXP3 (red) and (**D**) Treg content in atherosclerotic lesions (*n* = 9–10). (**E**) FOXP3 and CD25 expression by splenic Tregs (*n* = 9) and as control, CD8^+^ T cells (gray). (**F**) Cytokine secretion as well as (**G**) suppressive and (**H**) proliferative capacities of splenic CD4^+^CD25^+^ Tregs isolated from unchallenged PI3Kδ^+/+^ and PI3Kδ^–/–^ mice cultured (**F**) with anti-CD3/CD28–coated beads in the presence/absence of IL-2 (2,000 U/mL) or normal medium for 72 hours (*n* = 4), (**G**) at indicated ratios relative to PI3Kδ^+/+^ CD4^+^CD62L^+^ responder T (Tresp) cells in the presence of APCs and anti-CD3 antibody for 90 hours, and (**H**) with anti-CD3/CD28–coated beads in the presence/absence of indicated IL-2 levels for 85 hours. Data are representative of 3 experiments performed in triplicates (**G** and **H**). Suppression of Tresp cell proliferation as well as proliferation of Tregs (%) was measured and calculated, respectively. (**I** and **J**) Atherosclerotic lesion size in the whole aorta of indicated and PI3Kδ^–/–^→*Ldlr*^–/–^ male mice adoptively transferred with 10^6^ PI3Kδ^+/+^ (green) and PI3Kδ^–/–^ (purple) CD4^+^CD25^+^ Tregs at the start of the 6-week HFD period (*n* = 13–14). Statistics were done using 2-tailed unpaired *t* test (**A**–**F**) or 1-way ANOVA with Bonferroni’s post hoc test (**G**–**J**). *P < 0.05, **P < 0.01, ***P < 0.001, ****P < 0.0001.

As demonstrated, augmented atherogenesis in PI3Kδ^–/–^→*Ldlr*^–/–^ mice was characterized by diminished expression of atheroprotective cytokines IL-10 and IL-35 (*Ebi3*) in aortas expressed by various cells, including macrophages, B cells, and Tregs. Given that Tregs are protective against atherosclerosis (6, 8–10), we addressed a plausible role of Tregs in more detail. Tregs were decreased in LNs, spleen, and atheromata of PI3Kδ^–/–^→*Ldlr*^–/–^ mice compared with controls (Figure 6, A–D), which is in accordance with diminished peripheral Tregs in PI3Kδ^–/–^ mice at baseline (Supplemental Figure 6A). While splenic Tregs from PI3Kδ^–/–^→*Ldlr*^–/–^ mice maintained high IL-2Rα chain (CD25) expression as a feature of Tregs, they displayed reduced levels of the lineage-defining Forkhead box P3 (FOXP3) transcription factor (Figure 6E), whose steady high expression is essential for Treg development, stability, and suppressive function (30–32). Likewise, splenic Tregs from unchallenged PI3Kδ^–/–^ mice displayed decreased FOXP3 protein, albeit at different levels (Supplemental Figure 6B). Moreover, PI3Kδ^–/–^ Tregs isolated from unchallenged mice secreted reduced levels of their key atheroprotective cytokines IL-10 and TGF-β upon in vitro stimulation and exhibited an impaired suppression of CD4^+^ T cell proliferation (Figure 6, F and G, and Supplemental Figure 6C). Additionally, PI3Kδ^–/–^ Tregs displayed reduced proliferative capacities even in the presence of high IL-2 levels (Figure 6H and Supplemental Figure 6D). Thus, the lack of PI3Kδ signaling impairs numbers, functions, and stability of Tregs and may therefore lead to increased vascular inflammation and atherosclerosis.

We next investigated a potential causal connection between impaired Tregs and accelerated atherogenesis. Adoptive transfer of PI3Kδ^+/+^ CD4^+^CD25^+^ Tregs (Supplemental Figure 7A) into PI3Kδ^–/–^→*Ldlr*^–/–^ mice at the start of the HFD period completely blunted the atherosclerotic phenotype as it resulted in a plaque burden similar to that of PI3Kδ^+/+^→*Ldlr*^–/–^ controls (Figure 6, I and J), without affecting body weight and serum lipid levels (Supplemental Figure 7B). In contrast, adoptively transferred PI3Kδ^–/–^ CD4^+^CD25^+^ Tregs were unable to attenuate atherosclerosis (Figure 6, I and J), strongly suggesting that PI3Kδ deficiency specifically in Tregs aggravates atherosclerotic lesion formation.

**Figure 7:**
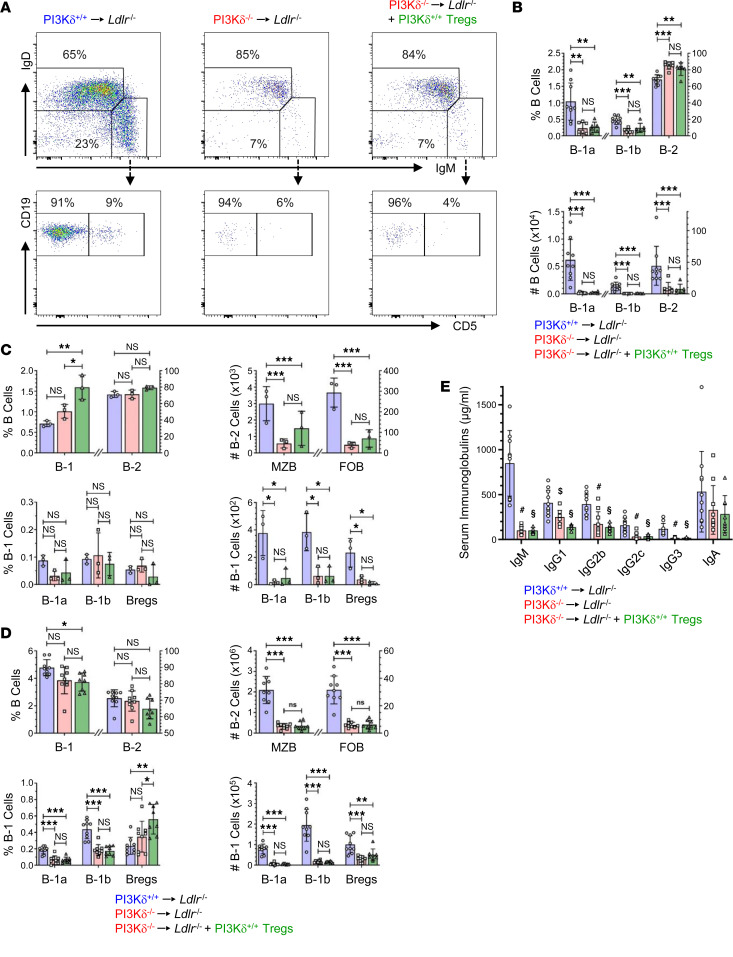
PI3Kδ^–/–^→*Ldlr*^–/–^ mice possess impaired mature B cells and serum Ig levels unaffected by adoptive transfer of PI3Kδ^+/+^ Tregs. (**A**) IgD, IgM, and CD5 expression of live pregated CD19^+^ cells as well as (**B**) proportion and number of IgM^hi^IgD^lo^CD5^+^ B-1a, IgM^hi^IgD^lo^CD5^–^ B-1b, and IgM^lo^IgD^hi^ B-2 cells in the PerC of indicated and PI3Kδ^–/–^→*Ldlr*^–/–^ male mice adoptively transferred with 10^6^ PI3Kδ^+/+^ (green) CD4^+^CD25^+^ Tregs (*n* = 6–9). Proportion and number of B-1, B-2, marginal zone B-2 (MZB), follicular B-2 (FOB), B-1a, B-1b, and regulatory B (Bregs) cells in (**C**) LNs and (**D**) spleen based on the gating strategy depicted in Supplemental Figure 8 (*n* = 3 LNs pooled of 3 mice each, *n* = 8–9 spleen). (**E**) Serum levels of depicted Ig isotypes from indicated male mice (*n* = 8–11). Statistics were done using 1-way ANOVA with Bonferroni’s post hoc test. *P < 0.05; **P < 0.01, ***P < 0.001. For **E**, ^#^**A** vs. **B**, *P* < 0.001; ^$^**A** vs. **B**, *P* < 0.01; **^§^A** vs. **C**, *P* < 0.001; all **B** vs. **C** were not significant.

Since B cells contribute to atherogenesis (6, 13, 14), and Tregs participate in the control of B cell responses (10, 13, 33), we finally analyzed mature B cell subsets and immunoglobulin levels more precisely. PI3Kδ^–/–^→*Ldlr*^–/–^ mice displayed diminished B-1 and B-2 cells in PerC (Figure 7, A and B), as well as impaired B-1 cells, marginal zone B (MZB) cells, and follicular B-2 (FOB) cells in LNs and spleen compared with PI3Kδ^+/+^→*Ldlr*^–/–^ controls (Figure 7, C and D, and Supplemental Figure 8), respectively. Accordingly, PI3Kδ^–/–^→*Ldlr*^–/–^ mice showed reduced levels of circulating IgM and IgG antibodies (Figure 7E). However, neither B cell numbers nor serum immunoglobulin levels of PI3Kδ^–/–^→*Ldlr*^–/–^ mice were affected by adoptively transferred PI3Kδ^+/+^ CD4^+^CD25^+^ Tregs (Figure 7, A–E). Although our data cannot exclude the possibility that impaired atheroprotective B-1 cells and serum IgM in particular contribute to augmented atherogenesis, such effects would then be blunted by fully functional Tregs.

## Discussion

Even though distinct T and B cell subsets possess exacerbating or inhibitory properties on the initiation and progression of atherosclerosis, analyses of mice deficient of mature lymphocytes demonstrated that adaptive immunity on balance is a key driver of atherosclerosis (1, 4–6). Since PI3Kδ is a central signaling molecule critically involved in a broad range of lymphocyte functions including activation, differentiation, and effector function of Th1 cells (15, 16), we aimed to target maladaptive pro-inflammatory immune responses through inactivation of hematopoietic PI3Kδ in order to attenuate atherosclerotic plaque progression. Consistent with our initial hypothesis, PI3Kδ inactivation had extensive effects on the adaptive immune system during atherogenesis, as demonstrated herein. PI3Kδ^–/–^→*Ldlr*^–/–^ mice were characterized by diminished numbers of peripheral T cells as well as impaired CD4^+^ T cell activation, Teff cell differentiation, and pro-inflammatory Th1 response. Moreover, they displayed reduced B cells and serum immunoglobulins. These data are in line with earlier studies of PI3Kδ mutant mice, demonstrating that PI3Kδ signaling is required for activation and differentiation of CD4^+^ Th cells as well as development and function of mature B cells (15–21).

However, although adaptive immunity was severely impaired, hematopoietic PI3Kδ deficiency unexpectedly led to aggravated atherosclerosis in male but not female *Ldlr*^–/–^ mice. Importantly, even PI3Kδ^+/+^→*Ldlr*^–/–^ female mice displayed plaque levels typically seen in PI3Kδ^–/–^→*Ldlr*^–/–^ male mice. This is in agreement with multiple previous studies showing that female mice develop greater plaque burden than males (34). The absence of any effect of hematopoietic PI3Kδ deficiency on plaque size in female mice suggests that lack of PI3Kδ exacerbates atherosclerosis only up to a certain threshold, which was already reached in PI3Kδ^+/+^→*Ldlr*^–/–^ female mice, and therefore no measurable effects were observed in this group. However, the mechanisms underlying sex as a biological variable in our atherosclerosis study remain elusive.

Aggravated atherosclerosis in male PI3Kδ^–/–^→*Ldlr*^–/–^ mice was associated with hampered numbers and stability of Tregs. Moreover, PI3Kδ^–/–^ Tregs possessed impaired immunosuppressive functions. Importantly, adoptive transfer of PI3Kδ^+/+^ Tregs into PI3Kδ^–/–^→*Ldlr*^–/–^ mice completely restrained accelerated atherosclerosis, whereas PI3Kδ^–/–^ Tregs failed to disburden these mice from aggravated lesion development. Overall, our data show that PI3Kδ deficiency impairs numbers, functions, and stability of Tregs and suggest that the lack of PI3Kδ signaling specifically in Tregs exacerbates atherosclerotic lesion formation. However, our data do not exclude a possible role of other hematopoietic cells such as impaired atheroprotective B-1 cells during enhanced atherogenesis in PI3Kδ^–/–^→*Ldlr*^–/–^ mice.

Within this study, we demonstrated that PI3Kδ deficiency impairs numbers of, the secretion of signature atheroprotective cytokines IL-10 and TGF-β of, and the suppressive capacity of Tregs similar to data on PI3Kδ^D910A/D910A^ Tregs published previously (35–37). Moreover, we showed that PI3Kδ deficiency impairs proliferative capacities of Tregs even in the presence of high IL-2 levels, which likely contributes to reduced numbers of Tregs in vivo. In fact, PI3Kδ signaling plays a key role downstream of T cell activation via TCR, IL-2R, CD28, and ICOS (15, 37) that governs multiple aspects of Treg biology, including development, activation, lineage stability, proliferation, survival, metabolic, and suppressive functions of Tregs through largely unknown mechanisms (30, 38–41). Although our data do not fully rule out extrinsic effects of PI3Kδ inactivation on Treg biology, particularly our in vitro data on Tregs implicate Treg-intrinsic requirements for PI3Kδ signaling to generate complete immunosuppressive functions. Consistent with this, recent data show that Treg-specific PI3Kδ deficiency is sufficient to disrupt Treg-mediated immunosuppression and immune tolerance of cancer (42), demonstrating Treg-intrinsic requirements for proper PI3Kδ signaling.

Tregs play a critical role in protecting against and regression of atherosclerosis through regulatory effects on various cell types, including Teff cells, macrophages, and B cells, by cell contact–dependent mechanisms and production of immunosuppressive cytokines (6–10, 43). In line with this, aortas of PI3Kδ^–/–^→*Ldlr*^–/–^ mice were characterized by decreased Treg numbers as well as reduced expression of IL-10 and IL-35. Importantly, previous studies demonstrated that diminished IL-10, TGF-β, and IL-35 levels as well as diminished Tregs enhanced pro-atherogenic immune responses including antigen presentation, T cell activation, Th1 response, classically activated macrophage (M1) polarization, and macrophage accumulation (6, 10, 44). While these pro-atherogenic effects of diminished Tregs and atheroprotective cytokines are well established, we herein present data demonstrating that impaired Tregs promoted plaque growth even on the background of an attenuated peripheral Th1 response. Indeed, we detected increased expression of pro-atherogenic IFN-γ, IL-1β, and CCL5 in atherosclerotic aortas of PI3Kδ^–/–^→*Ldlr*^–/–^ mice, which can be secreted by various cells including B cells, M1 macrophages, CD8^+^ T cells, and Th1 cells. Furthermore, expression of pro-atherogenic IL-12, TNF-α, and Th1 transcription factor T-bet was not decreased in atherosclerotic aortas as expected but remained unaltered or even showed a trend toward augmentation. However, these findings do not necessarily indicate a restored Th1 response as these markers are principally expressed by multiple subsets of immune and nonimmune cells (45). Interestingly, recent data suggest that T-bet–expressing invariant natural killer T (iNKT) cells and type I innate lymphoid cells (ILCs) aggravate atherosclerosis through the elaboration of pro-inflammatory cytokines IFN-γ and TNF-α (6, 7, 46), while Tregs control the activation of NKT cells and ILCs (47, 48). Given the impaired Th1 immunity and Treg response in PI3Kδ^–/–^→*Ldlr*^–/–^ mice, it remains an intriguing possibility that innate sources of IFN-γ and TNF-α from iNKT cells and type I ILCs may substantially contribute to augmented lesion development possibly through macrophage stimulation and M1 polarization.

In this context, it is noteworthy that cell-autonomous PI3Kδ deficiency altered crucial macrophage functions not at all or only slightly, including macrophage polarization as demonstrated herein. Therefore, it can be assumed that PI3Kδ-deficient macrophages are largely unrestricted in their response to changes in the local cytokine milieu that favored M1 polarization in PI3Kδ^–/–^→*Ldlr*^–/–^ mice. Because of M1 polarization and possibly activation of other CCL5-expressing cells, such as CD8^+^ T cells and vascular smooth muscle cells, CCL5 is increased in these mice. CCL5 mediates migration of Ly6C^hi^ monocytes, pro-atherogenic CCR5/FOXP3/IFN-γ–expressing CD4^+^ T cells, and possibly CCR5-expressing Th1 cells into atherosclerotic plaques (7, 11, 28, 29, 49). Therefore, one might speculate that an impaired Treg response in PI3Kδ^–/–^→*Ldlr*^–/–^ mice favored M1 macrophage polarization and thus increased CCL5 levels contributing to augmented plaque growth possibly by increased monocyte recruitment. Correspondent augmentation of IFN-γ and IL-1β expression in plaques might have propagated lesion growth by reinforcing these inflammatory mechanisms as shown elsewhere (1, 6).

In addition to Th1 and macrophage functions, Tregs control B cell responses through suppression of Th cells, B cell activation, proliferation, and antibody production (10, 13, 33). B cells are implicated in the regulation of atherosclerosis and their effects are subset specific. Innate-like B-1 and MZB cells protect against atherosclerosis through different mechanisms, including secretion of oxidized LDL–specific (oxLDL-specific) natural IgM antibodies as well as control of Th cell and germinal center responses (13, 14, 50). On the other hand, conventional FOB cells promote atherogenesis through stimulation of Th cell responses, secretion of IgG antibodies, and IgG-oxLDL immune complex–mediated M1 responses (13, 14, 51). Numbers and functions of these B cell subsets were reduced in PI3Kδ^–/–^→*Ldlr*^–/–^ mice. Thus, reduction of B-1 cells, MZB cells, and serum IgM might have contributed to enhanced lesion formation in PI3Kδ^–/–^→*Ldlr*^–/–^ mice. However, diminished FOB cells and serum IgG might have simultaneously limited atherosclerosis. Hence, our findings cannot exclude a possible role of impaired atheroprotective B-1 cells and serum IgM during intensified lesion development in PI3Kδ^–/–^→*Ldlr*^–/–^ mice. However, since adoptive transfer of PI3Kδ^+/+^ Tregs completely restrained aggravated atherosclerosis, our data demonstrate that potential effects of impaired B cell responses can be rescued by fully functional Tregs.

Our investigation has potential limitations. This study was largely based on BM-transplanted *Ldlr*^–/–^ mice, which are commonly used to study the participation of hematopoietic cells in atherosclerosis (52). However, irradiated *Ldlr*^–/–^ mice develop larger lesions within aortic roots and yet develop smaller lesions at the thoracic aorta (53). Thus, effects of hematopoietic PI3Kδ deficiency on atherosclerosis may be simultaneously influenced by effects of irradiation on vascular cells.

Collectively, our data demonstrate that the dominant effect of hematopoietic PI3Kδ during atherogenesis is to ensure proper Treg homeostasis, stability, and function in order to suppress pro-inflammatory immune responses and plaque progression. We identified PI3Kδ as a crucial driver of atheroprotective Treg responses, which outweigh pro-atherogenic effects exerted by Th1 cells. Consequently, Treg disturbance entails a switch to an augmented pro-inflammatory state in the local milieu of aortas and therefore promotes plaque growth even in the presence of a concomitant disturbed Th1 response. These findings improve our understanding of PI3Kδ signaling in Treg biology and plaque pathobiology, emphasizing the prominent role of Tregs in atherosclerosis. Thereby, fine-tuning PI3Kδ signaling in Tregs may represent a future therapeutic strategy.

## Methods

### Sex as a biological variable.

In our study, male and female mice were examined, and sex-dimorphic effects for atherogenic plaque burden were reported. Male mice were used exclusively for immunological studies to achieve less phenotype variability.

### Mice, BM transplantation, and metabolic characterization.

PI3Kδ^–/–^ mice (20) on a C57BL/6J background were provided by Roland Piekorz (Institute of Biochemistry and Molecular Biology II, Medical Faculty and University Hospital Düsseldorf, Heinrich Heine University, Düsseldorf, Germany). PI3Kδ^–/–^ mice were bred with C57BL/6J mice (Jackson Laboratory) followed by breeding the PI3Kδ^+/–^ offspring in order to generate PI3Kδ^–/–^ mice and PI3Kδ^+/+^ littermate controls. For BM transplantation, 8-week-old B6.129S7-*Ldlr^tm1Her^*/J (*Ldlr*^–/–^) mice (27) (Jackson Laboratory) and CD45.1-expressing B6.SJL-*Ptprc^a^ Pepc^b^*/BoyCrl mice (Charles River Laboratories) were lethally irradiated with 10 Gy from a linear accelerator followed by intravenous injection with 5 × 10^6^ BM cells isolated from femurs of PI3Kδ^–/–^ and PI3Kδ^+/+^ mice at 24 hours after irradiation. Recipient mice were kept on a 12-hour light/12-hour dark cycle and were fed an HFD containing 15.8% fat and 1.25% cholesterol (TD.94059, Altromin) ad libitum for 6 weeks, beginning at 12 weeks of age. At the end of the study, mice were fasted overnight and weighed. Finally, mice were anesthetized by inhalation of 2%–3% isoflurane and euthanized via cardiac exsanguination or cervical dislocation wherever appropriate. Blood samples were collected from the left ventricle for cellular, Ig isotype, and lipid analyses. Lipid levels were determined in serum with a Roche P800 modular analyzer. Blood glucose levels were measured with a GlucoMen Glycó system (Menarini Diagnostics).

### PCR analysis of donor BM reconstitution.

Efficient donor BM reconstitution was verified by PCR amplification of PI3Kδ^–/–^ and PI3Kδ^+/+^ DNA alleles from peripheral blood of HFD-fed mice using GoTaq DNA Polymerase (Promega) and gene-specific primers (*Pik3cd*-5′, 5′-TGAGATGCTGTGCAAGACTGTG-3′; *Pik3cd*^+/+^-3′, 5′-AATAGCATGAGGTTGGCCCAAG-3′; *Pik3cd*^–/–^-3′, 5′-AATAGCATGAGGTTGGCCCAAG-3′).

### Atherosclerotic lesion analysis and immunohistochemistry.

After 6 weeks on HFD, mice were sacrificed, and the aortic tree was perfused with 0.7% NaCl. The heart and whole aorta including the iliac bifurcation was dissected en bloc; minor branching arteries and fat tissue were carefully removed. For en face aorta analyses, the aorta was dissected from the heart and fixed in 4% paraformaldehyde/PBS for 12 hours, and the aortic lumen was opened with a longitudinal incision. The opened aorta was pinned onto a silicon plate and stained for lipids by 0.5% Sudan IV for 15 minutes according to standard protocols. Images of the aorta were obtained. The area of aortic lesions and percentage of aortic area stained with Sudan IV was determined using ImageJ (NIH) and Photoshop (Adobe) software. The heart was frozen in OCT embedding medium (Sakura Finetek) on isopentane. Sequential 7 μm sections were cut out of the heart where the atrioventricular valves were visible. For lesion area measurements, 4 Oil Red O–stained sections starting at first appearance of the valve leaflets with an interval of 42 μm were analyzed with ImageJ and Photoshop software. In addition, randomly selected sections from within the same area used for atherosclerotic lesion analysis were stained after fixation with ice-cold acetone by incubation with rat anti-mouse CD4 (RM4-5, eBioscience), FOXP3 (FJK-16s, eBioscience), or MOMA-2 (MCA519G, AbD Serotec) antibodies followed by the SuperVision2AP-Polymer system (DCS-Diagnostics) and counterstaining of cell nuclei with hematoxylin.

### qRT-PCR.

Total RNA from aortas was isolated with TRIzol reagent (Invitrogen) and RNeasy columns (QIAGEN). cDNA was prepared with SuperScript III first-strand synthesis system (Invitrogen). qRT-PCR of *Il1b*, *Il4*, *Il10*, *Il12a*, *Il17a*, *Ifng*, *Tnf*, *Tgfb1*, *Tbx21*, *Gata3*, *Rorc*, *Ebi3*, *Csf1*, *Ccl2*, *Ccl5*, *Cxcl1*, *Cx3cl1*, *Mif*, and *Gapdh* genes was performed using TaqMan gene expression assays (Applied Biosystems).

### Flow cytometry.

Peripheral blood mononuclear cells, splenocytes, para-aortic lymph node cells, and peritoneal cells were stained after blocking of Fcγ receptors using a rat anti-mouse CD16/CD32 antibody (2.4G2) with combinations of fluorochrome-conjugated monoclonal antibodies against CD3ε (145-2C11), CD4 (RM4-5), CD8α (53-6.7), CD11b (M1/70), CD25 (PC61), CD43 (S7), CD69 (H1.2F3), Ly-6G (1A8), IFN-γ (XMG1.2; all from BD Biosciences), NK1.1 (PK136), TER-119 (TER-119), CD19 (6D5), CD44 (IM7), F4/80 (BM8), CD115 (AFS98), Ly-6C (HK1.4), CCR1 (S15040E), CCR2 (SA203G11), CCR5 (HM-CCR5), CXCR2 (SA045E1), CX3CR1 (SA011F11), IL-4 (11B11), IgD (11-26c.2a), CD45.1 (A20), CD45.2 (104; all from BioLegend), FOXP3 (FJK-16s), IgM (II/41), CD62L (MEL-14), and CD5 (53-7.3; all from eBioscience) in HBSS medium (with 3% FCS/25 mM HEPES/1 mM EDTA/0.1% NaN_3_). Intracellular FOXP3 staining was performed according to manufacturers’ protocols. For intracellular staining of cytokines, cells were cultured in RPMI-1640 medium (with GlutaMAX/10 mM HEPES/1 mM sodium pyruvate/100 U/mL penicillin/100 μg/mL streptomycin/50 μM 2-ME/10% FCS) at a density of 1 × 10^6^ cells/mL for 5 hours with 5 ng/mL phorbol-12-myristate 13-acetate and 500 ng/mL ionomycin (both from MilliporeSigma) in the presence of 2 μM Monensin (BD Biosciences). After staining cell surface antigens, intracellular cytokines IFN-γ and IL-4 were determined using the Cytofix/Cytoperm Fixation/Permeabilization Kit (BD Biosciences) according to manufacturers’ protocols. Cells were analyzed on a FACSCanto II (BD Biosciences) and FlowJo software (Tree Star).

### Treg isolation, transfer, suppression, and proliferation assays, as well as T cell cytokine secretion.

Tregs were isolated from splenocytes with the CD4^+^CD25^+^ Regulatory T Cell Isolation Kit (Miltenyi Biotec). A total of 10^6^ CD4^+^CD25^+^ Tregs from unchallenged PI3Kδ^+/+^ and PI3Kδ^–/–^ mice were transferred intravenously into PI3Kδ^–/–^ transplanted *Ldlr*^–/–^ mice at the start of the 6-week HFD, respectively. For Treg suppression assays, 10^5^ naive PI3Kδ^+/+^ CD4^+^CD62L^+^ Tresp cells labeled with 5 μM CFSE (Invitrogen) were cultured with 10^5^, 5 × 10^4^, 2.5 × 10^4^, 1.25 × 10^4^, and 6.25 × 10^3^ PI3Kδ^+/+^ or PI3Kδ^–/–^ Tregs in the presence of 2.0 μg/mL anti-CD3ε antibody (145-2C11, eBioscience) on 5 × 10^5^ T cell–depleted PI3Kδ^+/+^ antigen-presenting cells (3 Gy irradiated) for 90 hours. Proliferation of CFSE-labeled Tresp cells was analyzed by flow cytometry after exclusion of dead cells using 7-AAD. For the determination of secreted cytokines and proliferation, splenic CD4^+^CD25^+^ Tregs and CD4^+^CD25^–^ T cells were isolated via the CD4^+^ T Cell Isolation Kit (Miltenyi Biotec) followed by cell sorting using a FACSAria III (BD Biosciences) with a final purity of at least 98%. A total of 10^5^ CD4^+^CD25^+^ Tregs and CD4^+^CD25^–^ T cells were cultured in 100 μL RPMI-1640 medium (with GlutaMAX/10 mM HEPES/1 mM sodium pyruvate/100 U/mL penicillin/100 μg/mL streptomycin/50 μM 2-ME/10% FCS or, for TGF-β1 assays, 1% Nutridoma SP [Roche] instead of FCS) with or without 3 × 10^5^ anti-CD3/CD28 preloaded MACSiBeads and 2,000 U/mL rIL-2 (Miltenyi Biotec) for 72 hours. For proliferation assays, 5 × 10^4^ CD4^+^CD25^+^CD62L^+^ naive Tregs labeled with 1 μM CFSE were cultured in 200 μL RPMI-1640 medium with 1.5 × 10^5^ anti-CD3/CD28 preloaded MACSiBeads in the presence or absence of 500, 1,000, and 2,000 U/mL rIL-2 (Miltenyi Biotec) for 86 hours.

### Multiplexing.

IL-1β, IL-4, IL-6, IL-10, IL-12p70, IFN-γ, TNF-α, CCL2, and CCL5 secreted into culture medium was measured with ProcartaPlex multiplex immunoassays (Invitrogen) on a Luminex system.

### Serum Ig levels.

Serum antibody levels were determined using Legendplex mouse immunoglobulin isotyping multiplex immunoassays (BioLegend).

### Isolation and culture of macrophages.

For preparation of BMDMs, BM cells were subjected to red blood cell lysis and plated on Petri dishes in RPMI-1640 medium (with GlutaMAX/10% FCS/1 mM sodium pyruvate/100 U/mL penicillin/100 μg/mL streptomycin/20% L929 [MilliporeSigma] conditioned medium). At day 8 of culture, BMDMs were plated on tissue culture dishes for 2 more days of culture before the start of experiments. To isolate PerM, mice were injected with 1 mL 2.5% thioglycollate medium (BD Biosciences) intraperitoneally. At 4 days later, peritoneal cells were collected with ice-cold PBS and plated at a concentration of 10^6^ cells/mL in RPMI-1640 medium with 10% FCS. After 3 hours, nonadherent cells were removed by washing with PBS. Assays were performed 2 days upon original plating. Flow cytometry revealed that at least 97% of all cells expressed the F4/80 macrophage antigen.

### Foam cell formation assay.

After 24 hours’ starvation in RPMI-1640 medium with 0.5% FCS, PerM were cultured in RPMI-1640 medium containing 2% lipoprotein-depleted FCS in the presence or absence of 50 μg/mL and 100 μg/mL medium oxLDL (Kalen Biomedical) for 48 hours. Thereafter, cells were washed 3 times with PBS and fixed in 4% paraformaldehyde for 20 minutes. Neutral lipids were stained by using the BODIPY 493/503 lipid probe (Invitrogen) according to manufacturer’ s instructions followed by counterstaining of cell nuclei with DAPI. Cells that contained more than 3 clear BODIPY-labeled lipid droplets were considered foam cells.

### Efferocytosis assay.

Jurkat T cells (MilliporeSigma) were labeled with 5 μM Calcein-AM (eBioscience) according to manufacturer’s instructions followed by apoptosis induction by UVB radiation (450 mJ/cm^2^). Flow cytometry–verified, apoptotic, Calcein-AM–labeled Jurkat T cells were extensively washed with macrophage medium, and 10^6^ or 3 × 10^6^ cells were plated on top of 10^6^ PerM for 30 or 60 minutes at 37°C. After removing excessive apoptotic Jurkat T cells by extensive washes, fixed PerM (Cytofix/Cytoperm Kit, BD Biosciences) were stained with a rat anti-mouse F4/80-APC antibody (BM8, BioLegend). Efferocytosis was evaluated by the percentage of F4/80^+^ macrophages that were also Calcein-AM^+^ using a FACSCanto II (BD Biosciences). Under the conditions used, no significant basal adherence of Jurkat T cells to macrophages was observed.

### Macrophage polarization.

For M1 polarization, 10^6^ BMDMs were stimulated with 100 ng/mL IFN-γ (BioLegend) and 1 μg/mL LPS (MilliporeSigma) for 24 hours in 1 mL RPMI-1640 medium with 10% FCS on a 6-well plate. For M2 polarization, BMDMs were preincubated for 12 hours with 50 ng/mL IL-6 followed by stimulation with 10 ng/mL IL-4 and 50 ng/mL IL-6 (BioLegend) for 24 hours. As controls, macrophages were cultured with the respective medium alone.

### Statistics.

Data are presented as individual dot plots with bars denoting the mean ± SD. While statistical significance of pairwise comparisons of groups was determined using 2-tailed unpaired Student’s *t* test with Welch’s correction in case of unequal variances, 1-way ANOVA with Bonferroni’s post hoc test was used for multiple-group comparisons. Statistical analysis was performed using the GraphPad Prism 10 software. Probability values of *P* < 0.05 were considered significant.

### Study approval.

All animal experiments were approved by the district council of Cologne, Germany (approval number 81-02.04.2018.A186), and performed in accordance with German Laws for Animal Protection and guidelines from Directive 2010/63/EU of the European Parliament.

### Data availability.

All data are available in the Supporting Data Values file.

## Author contributions

MZ designed, performed, and supervised experiments; analyzed data; and wrote the manuscript. EMB, LGF, CM, FSRP, MK, SG, AP, LZ, RJN, and CV performed experiments and analyzed data. MP, SSP, and HW provided materials and instrumentation and contributed to experimental design and data analyses. SB acquired funding and contributed to experimental design. MV and SR acquired funding, designed experiments, and wrote the manuscript. All authors reviewed and commented on the manuscript.

## Supplementary Material

Supplemental data

Supporting data values

## Version 1 10/08/2024

In-Press Preview

## Version 2 11/22/2024

Electronic publication
