# Supplementary material for: Hematopoietic PI3Kδ deficiency aggravates murine atherosclerosis through impairment of Tregs
Source: JCI Insight. 2024 Nov 22;9(22):e155626. doi: 10.1172/jci.insight.155626 (PMC11601942; doi:10.1172/jci.insight.155626)
Supplement: Supplemental data [file jciinsight-9-155626-s131.pdf]

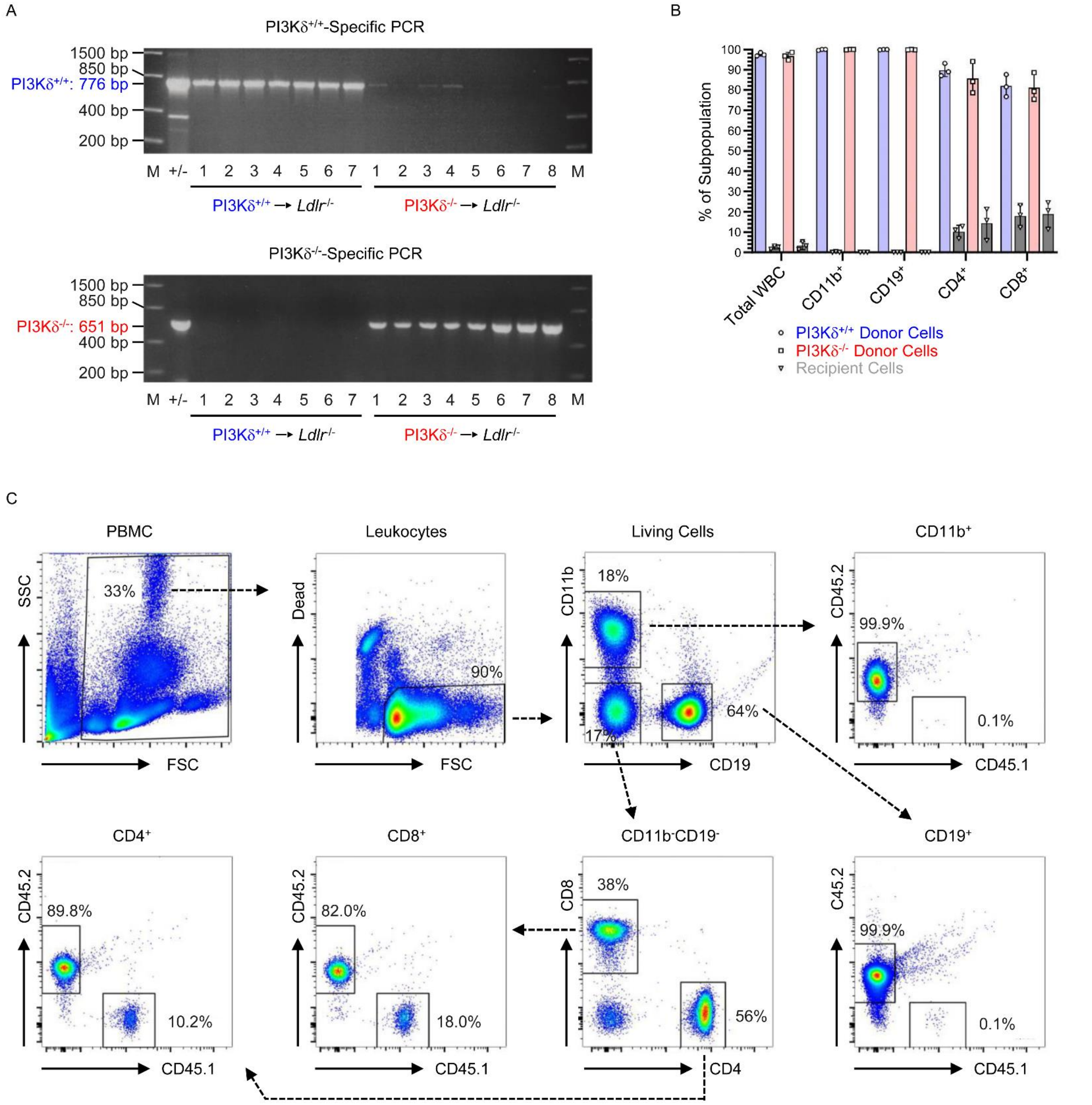

Supplemental Figure 1. Effective donor bone marrow reconstitution in PI3Kδ$^{-/-}$→*Ldlr*$^{-/-}$ mice. (**A**) Efficient donor bone marrow reconstitution in PI3Kδ$^{+/+}$→*Ldlr*$^{-/-}$ (blue) and PI3Kδ$^{-/-}$→*Ldlr*$^{-/-}$ mice (red) verified by PI3Kδ$^{+/+}$- and PI3Kδ$^{-/-}$-specific PCR amplification of blood DNA alleles. DNA from heterozygous PI3Kδ$^{+/-}$ mice (+/-) served as control. Length (bp, base pairs) of markers (M) and specifically amplified bands as indicated. (**B**) Homogeneous transplantation of CD45.2 PI3Kδ$^{+/+}$ (blue) and PI3Kδ$^{-/-}$ (red) bone marrow into CD45.1 mice (n = 3) reveals equally high proportions of donor-derived total white blood cells (WBC), CD11b^{+} myeloid cells, CD19^{+} B cells, as well as CD4^{+} and CD8^{+} T cells among peripheral blood mononuclear cells (PBMC) compared with low proportions of CD45.1 recipient cells (grey), based on the gating strategy depicted in (**C**). No significant differences in donor-derived immune cell-subsets were observed among groups. Statistics were done using 2-tailed unpaired *t*-test (**B**).

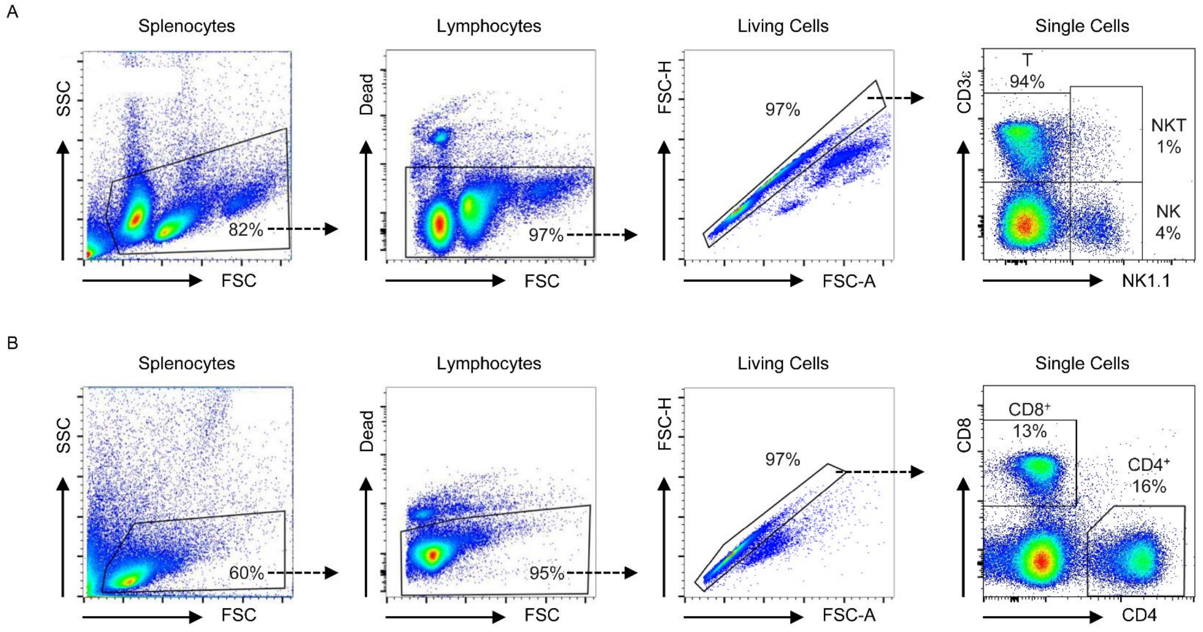

Supplemental Figure 2. Identification of peripheral T-cell subsets in mice. Gating strategy for (**A**) live $CD3^{+}NK1.1^{-}$ $CD4^{+}$ or $CD8^{+}$ T cells (depicted in Figure 1B, 2A, 2C, and Supplemental Figure 3A) and for (**B**) live $CD4^{+}$ or $CD8^{+}$ T cells (depicted in Figure 2E, 6A, and Supplemental Figure 3C) from LNs and spleen of mice.

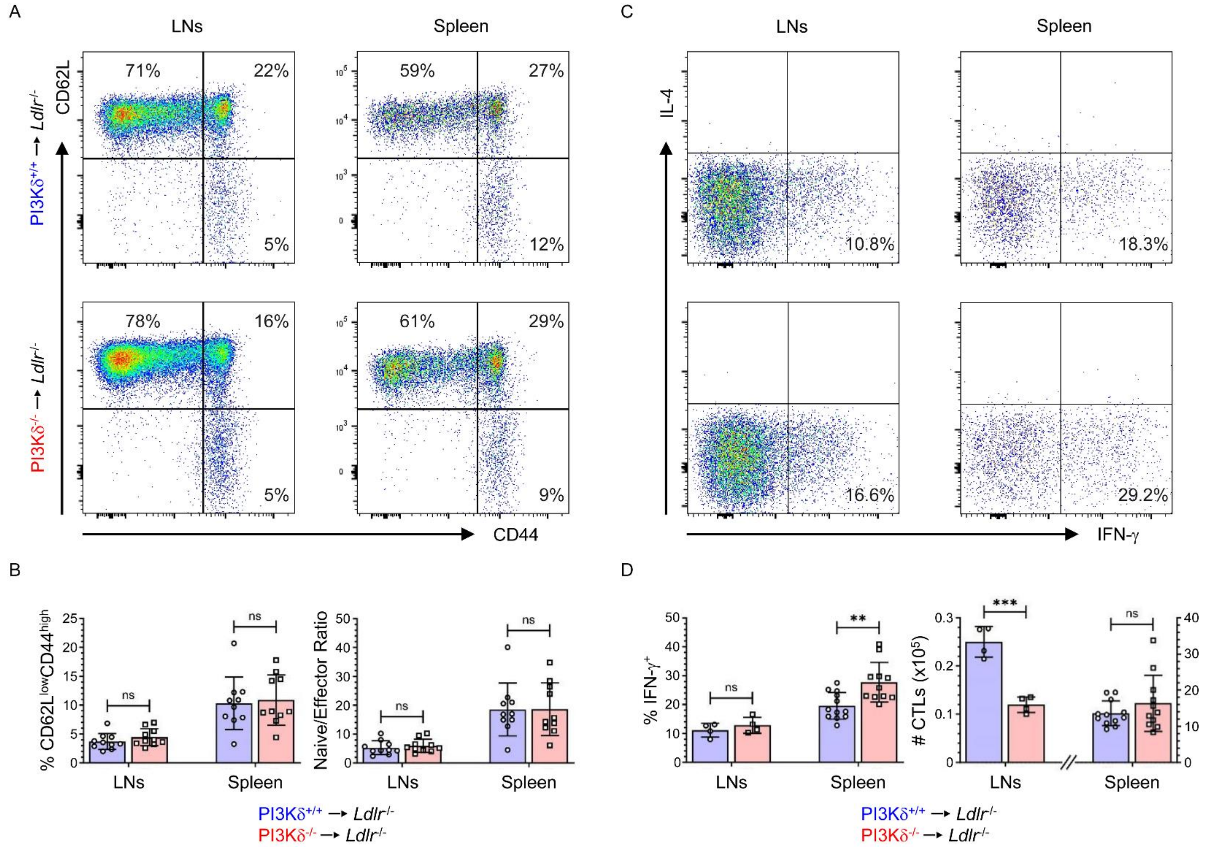

Supplemental Figure 3. PI3Kδ deficiency has limited impact on CD8⁺ Teff-cell differentiation and cytotoxic CD8⁺ T-cell response. Leukocytes from LNs and spleen of PI3Kδ$^{+/+}$→*Ldlr*$^{-/-}$ (blue) and PI3Kδ$^{-/-}$→*Ldlr*$^{-/-}$ mice (red) were pre-gated for live CD3^{+}NK1.1^{-}CD8^{+} T cells and investigated for (**A**) CD62L and CD44 expression, (**B**) proportion of CD62L^{low}CD44^{high} effector cells and quantification of naïve/effector CD8^{+} T cell ratio (n = 10), (**C**) intracellular expression of IFN-γ and IL-4 by pre-gated live CD8^{+} T cells, and (**D**) proportion and number of IFN-γ^{+} cytotoxic CD8^{+} T cells (CTLs) (n = 4 LNs pooled of 3 mice each, n = 11 - 12 spleen). Statistics were done using 2-tailed unpaired *t*-test. *P<0.05, **P<0.01, ***P<0.001; ns, not significant.

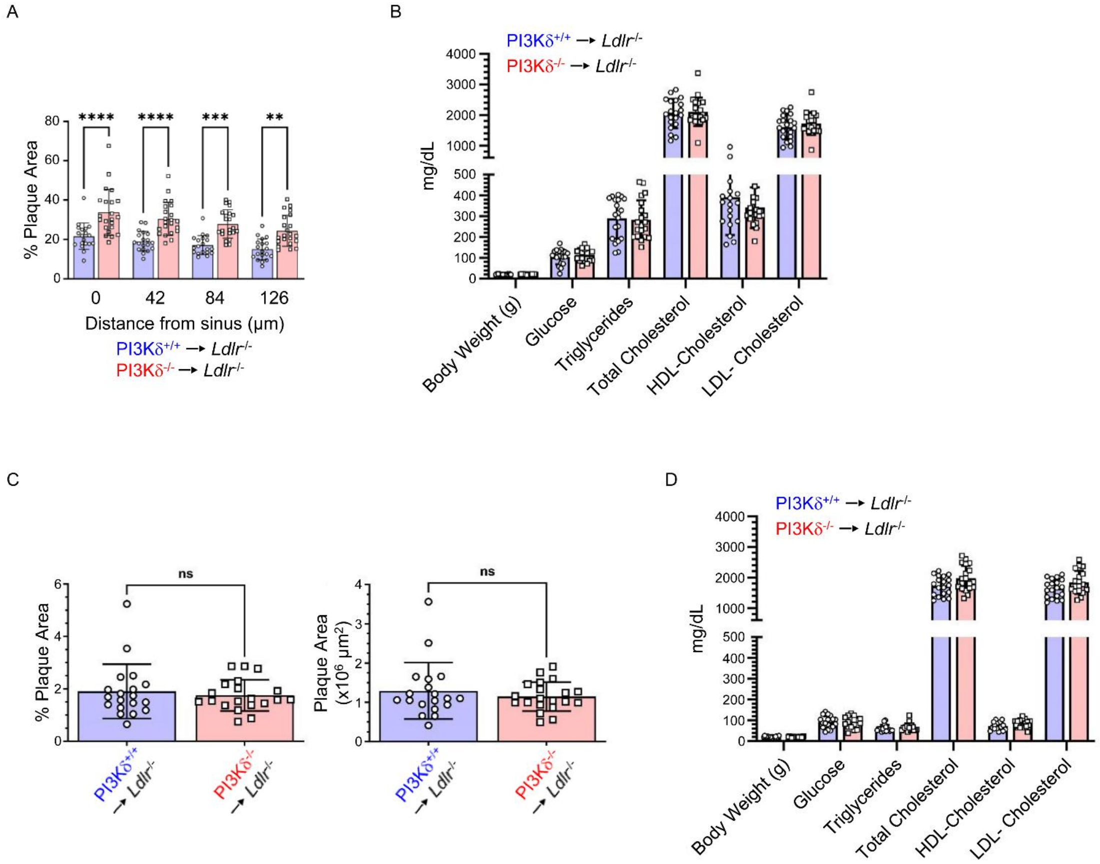

Supplemental Figure 4. Hematopoietic PI3Kδ deficiency aggravates atherosclerosis in male but not female *Ldlr*$^{-/-}$ mice. (**A**) Quantification of atherosclerotic lesion size depicted as relative plaque area (lesion area as % of total vessel area) in the aortic sinus of PI3Kδ$^{+/+}$→*Ldlr*$^{-/-}$ (blue) and PI3Kδ$^{-/-}$→*Ldlr*$^{-/-}$ male mice (red) at four analyzed levels with an interval of 42 µm starting at the first appearance of the leaflets of the heart with a distance of 0 µm (n = 19 – 22). (**B**) Body weight as well as fasting levels of glucose, triglyceride, total cholesterol, HDL-cholesterol, and LDL-cholesterol were comparable between groups of indicated male mice (n = 20). No significant differences in body weight, glucose, and lipids were observed among groups. (**C**) Quantification of atherosclerotic burden in the whole aorta of PI3Kδ$^{+/+}$→*Ldlr*$^{-/-}$ (blue) and PI3Kδ$^{-/-}$→*Ldlr*$^{-/-}$ (red) female mice depicted as relative plaque area (lesion area as % of total vessel area) and plaque area (µm^2), respectively. (n = 19 - 20). (**D**) Body weight as well as fasting levels of glucose, triglyceride, total cholesterol, HDL-cholesterol, and LDL-cholesterol were comparable between groups of indicated female mice (n = 19 – 20). No significant differences in body weight, glucose, and lipids were observed among groups. Statistics were done using 1-way ANOVA with Bonferroni's post hoc test (**A**) or 2-tailed unpaired *t*-test (**B-D**). **P*<0.05, ***P*<0.01, ****P*<0.001, *****P*<0.0001; ns, not significant.

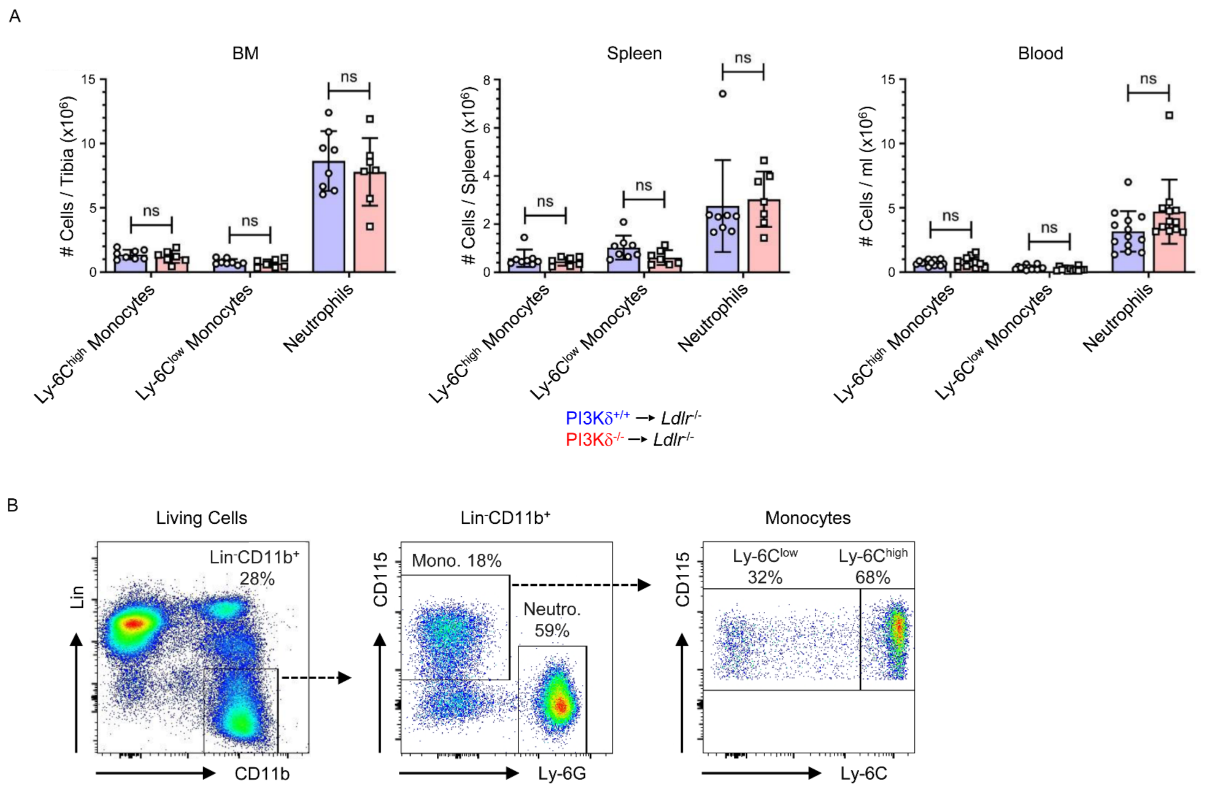

Supplemental Figure 5. Normal monocytes and neutrophils in PI3Kδ$^{-/-}$→*Ldlr*$^{-/-}$ mice. (**A**) Number of Ly-6C^{high} inflammatory monocytes, Ly-6C^{low} patrolling monocytes, and neutrophils in BM (n = 7 - 8), spleen (n = 7 – 8), and peripheral blood (n = 12 – 13) of PI3Kδ$^{+/+}$→*Ldlr*$^{-/-}$ (blue) and PI3Kδ$^{-/-}$→*Ldlr*$^{-/-}$ (red) male mice based on the gating strategy depicted in (**B**). Statistics were done using 2-tailed unpaired *t*-test. **P*<0.05, ***P*<0.01, ****P*<0.001; ns, not significant.

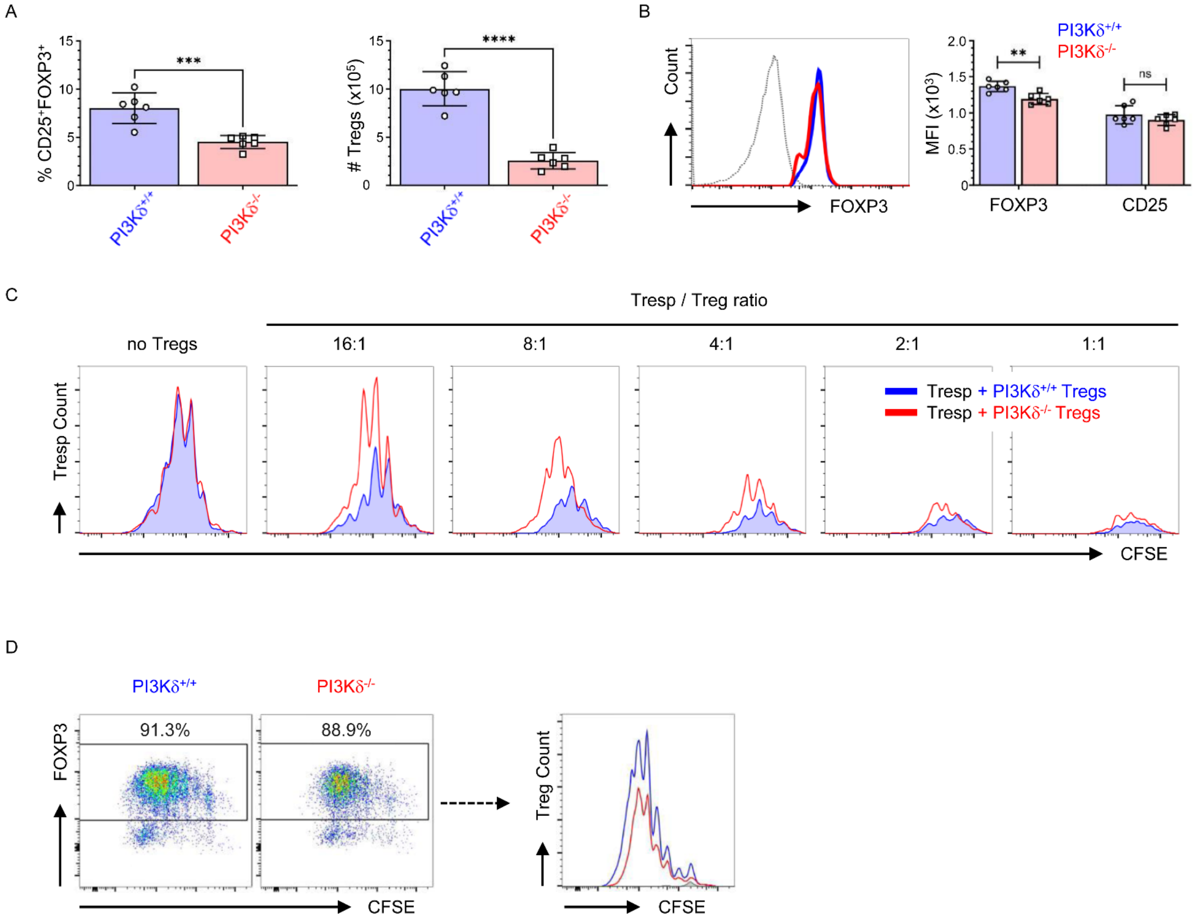

Supplemental Figure 6. Impaired numbers, stability, and functions of PI3Kδ$^{-/-}$ Tregs. (**A**) Proportion and number, as well as (**B**) FOXP3 and CD25 expression levels of splenic CD4$^+$CD25$^+$FOXP3$^+$ Tregs from unchallenged PI3Kδ$^{+/+}$ (blue) and PI3Kδ$^{-/-}$ mice (red) and as controls, CD8$^+$ T cells from PI3Kδ$^{-/-}$ mice (dotted gray line) (n = 6). (**C**) Proliferation of CFSE-labeled PI3Kδ$^{+/+}$ CD4$^+$ Tresp cells stimulated for 90 hours with anti-CD3 antibody in the presence of APCs and CD4$^+$CD25$^+$ Tregs from unchallenged PI3Kδ$^{-/-}$ or PI3Kδ$^{+/+}$ mice at various ratios (1:1 to 16:1) or without Tregs. Histograms display proliferation of Tresp cells cultured with PI3Kδ$^{+/+}$ Tregs (filled blue curves) or PI3Kδ$^{-/-}$ Tregs (red lines). (**D**) Proliferation of CFSE-labeled splenic CD4$^+$CD25$^+$CD62L$^+$ Tregs isolated from unchallenged PI3Kδ$^{+/+}$ and PI3Kδ$^{-/-}$ mice upon 85 hours of activation by anti-CD3/CD28-coated beads in the presence of 500 U/ml IL-2. Dot plots display proliferation and intracellular FOXP3 expression of pre-gated live CD4$^+$ T cells, and histograms depict proliferation of pre-gated live CD4$^+$FOXP3$^+$ cells. Data are representative of 3 experiments performed in triplicates (C-D). Statistics were done using 2-tailed unpaired *t*-test (**A-B**). *P<0.05, **P<0.01, ***P<0.001, ****P<0.0001; ns, not significant.

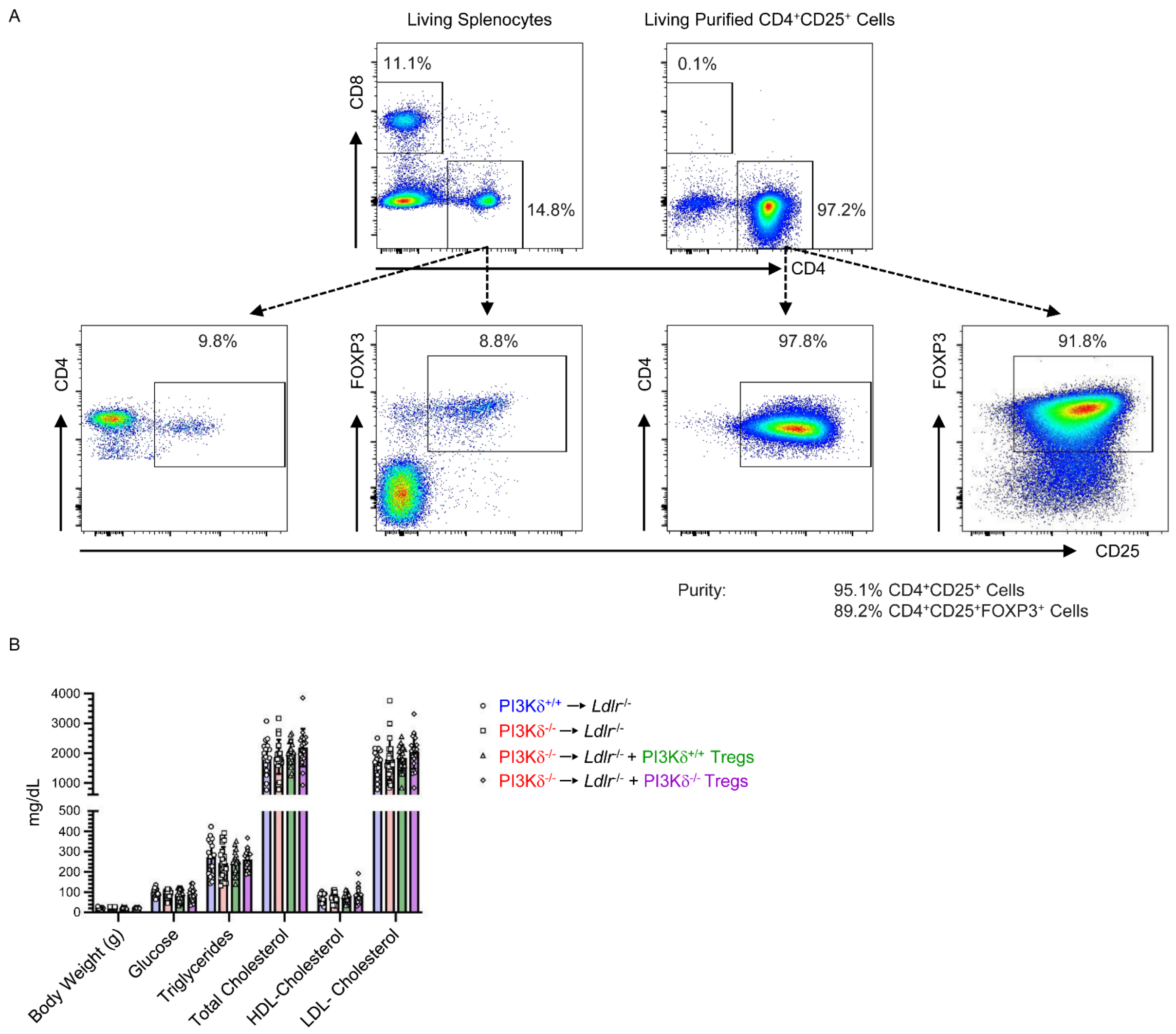

Supplemental Figure 7. Purity of isolated CD4$^+$CD25$^+$ Tregs and metabolic characterization of PI3Kδ$^{-/-}$→*Ldlr*$^{-/-}$ mice adoptively transferred with PI3Kδ$^{+/+}$ and PI3Kδ$^{-/-}$ Tregs. (**A**) Expression of CD4, CD8, CD25, and intracellular FOXP3 by living PI3Kδ$^{+/+}$ splenocytes and MACS-purified CD4$^+$CD25$^+$ cells. Adoptively transferred PI3Kδ$^{+/+}$ CD4$^+$CD25$^+$ cells were ~95% pure and ~89% were CD4$^+$CD25$^+$FOXP3$^+$ Tregs. (**B**) Body weight as well as fasting levels of glucose, triglyceride, total cholesterol, HDL-cholesterol, and LDL-cholesterol were comparable between groups of indicated and PI3Kδ$^{-/-}$→LDLR$^{-/-}$ male mice adoptively transferred with either 10^6 PI3Kδ$^{+/+}$ (green) or PI3Kδ$^{-/-}$ (purple) CD4$^+$CD25$^+$ Tregs at the start of the 6-weeks-HFD-period (n = 17 – 20). No significant differences in body weight, glucose, and lipids were observed among groups. Statistics were done using 1-way ANOVA with Bonferroni's post hoc test (**B**).

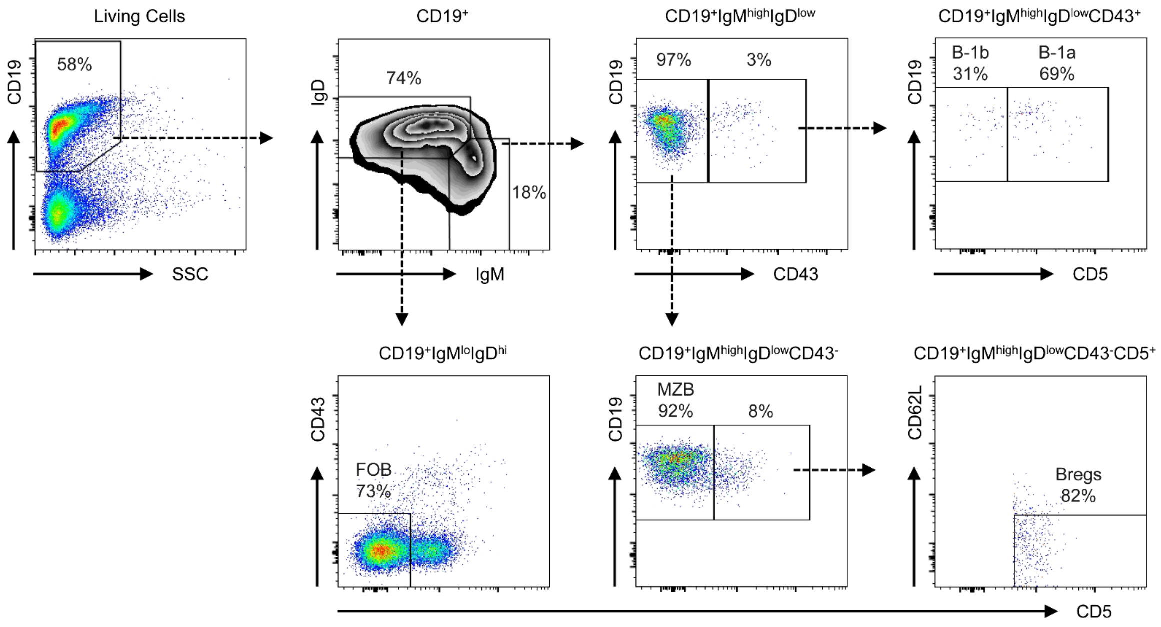

Identification of mature B-cell subsets in spleen and LNs based on Baumgarth (2011).

	B-1a cells	B-1b cells	Marginal Zone B-2 cells (MZB)	Follicular B-2 cells (FOB)	Regulatory B cells (Bregs)
Cell surface phenotype	$CD19^{high}$ IgM^{high} IgD^{low} $CD43^{+}$ $CD5^{+}$	$CD19^{high}$ IgM^{high} IgD^{low} $CD43^{+}$ $CD5^{-}$	$CD19^{mid}$ IgM^{high} IgD^{low} $CD43^{-}$ $CD5^{-}$	$CD19^{mid}$ IgM^{low} IgD^{high} $CD43^{-}$ $CD5^{-}$	$CD19^{high}$ IgM^{high} $IgD^{low/mid}$ $CD43^{-}$ $CD5^{+}$ $CD62L^{-}$

Baumgarth N.; Nat Rev Immunol. 2011 Jan;11(1):34-46.

Supplemental Figure 8. Gating strategy for the identification of mature $CD19^{+}$ B-cell subsets in LNs and spleen of mice. Leukocytes from LNs and spleen of mice were pre-gated for viable cells and $CD19^{+}SSC^{lo}$ B cells were analyzed for expression of surface IgM and IgD. Gated $CD19^{+}SSC^{lo}IgM^{lo}IgD^{hi}$ B cells were further analyzed for the expression of CD43 and CD5, identifying $CD19^{+}SSC^{lo}IgM^{lo}IgD^{hi}CD43^{-}CD5^{-}$ FOB cells. $CD19^{+}SSC^{lo}IgM^{hi}IgD^{lo}$ B cells were examined for CD43, CD5 and CD62L expression, distinguishing between $CD19^{+}SSC^{lo}IgM^{hi}IgD^{lo}CD43^{-}CD5^{-}$ MZB cells, $CD19^{+}SSC^{lo}IgM^{hi}IgD^{lo}CD43^{-}CD5^{+}CD62L^{-}$ Bregs, $CD19^{+}SSC^{lo}IgM^{hi}IgD^{lo}CD43^{+}CD5^{+}$ B-1a and $CD19^{+}SSC^{lo}IgM^{hi}IgD^{lo}CD43^{+}CD5^{-}$ B-1b cells.
